# The effectiveness of digital health intervention on glycemic control and physical activity in patients with type 2 diabetes: a systematic review and meta-analysis

**DOI:** 10.3389/fdgth.2025.1630588

**Published:** 2025-07-29

**Authors:** Haoyuan Xue, Lin Zhang, Yarong Shi, Hao Zhang, Chuanrong Zhang, Yang Liu, Wenshu Tan, Yaorong Liu

**Affiliations:** ^1^ School of Physical Education, Xi'an Physical Education University, Xi'an, China; ^2^ Informatization Construction Office, Xi'an Physical Education University, Xi'an, China; ^3^ School of Animation and Digital Arts, Shanxi Communication University, Taiyuan, China; ^4^ School of Wushu, Xi'an Physical Education University, Xi'an, China

**Keywords:** type 2 diabetes, physical activity, digital health intervention, glycemic control, meta-analysis

## Abstract

**Introduction:**

Digital health interventions (DHIs) offer promising strategies for managing type 2 diabetes mellitus (T2DM), yet their efficacy on physical activity remains inconsistent. This systematic review and meta-analysis evaluates DHIs' effectiveness across key clinical endpoints.

**Methods:**

Following PRISMA guidelines and PROSPERO registration (CRD420251032375), five databases (Web of Science, Embase, Scopus, Cochrane, PubMed) were searched through February 2025. Randomized controlled trials (RCTs) assessing DHIs (mobile applications, phone calls or SMS, online platforms, remote monitoring) versus usual care in T2DM patients were included. Primary outcomes were HbA1c, fasting blood glucose (FBG), postprandial blood glucose (PBG), HOMA-IR, and physical activity. Risk of bias was evaluated using Cochrane RoB 2. Meta-analyses employed random/fixed-effect models in Review Manager 5.3, with subgroup and sensitivity analyses for heterogeneity (*I*² > 50%).

**Results:**

From 9,499 records, 118 RCTs (21,662 participants) were analyzed. DHIs significantly reduced HbA1c (MD = −0.32% to −0.54%), FBG (MD = −0.30 to −0.85), and PBG (SMD = −0.58) versus controls (*p* < 0.05). Subgroup analyses indicated online platforms most effectively lowered HbA1c (MD = −0.54). No improvements occurred in HOMA-IR (MD = −0.18, 95% CI: −0.79 to 0.44) or physical activity (SMD = 0.16, 95% CI: −0.08 to 0.39). Cost analyses revealed lower expenses in DHI groups (mean: $269.31 vs. $465.37). High heterogeneity (*I*² = 69–92%) was observed for glycemic outcomes, partially explained by intervention duration and sample size in meta-regression.

**Discussion:**

DHIs demonstrate robust efficacy for glycemic management in T2DM, particularly through online platforms and remote monitoring. However, they fail to enhance physical activity or insulin resistance. Future studies should prioritize adaptive designs for sustained behavioral change and investigate long-term cost-effectiveness.

**Systematic Review Registration:**

https://www.crd.york.ac.uk/PROSPERO/view/CRD420251032375, identifier (CRD420251032375).

## 1 Introduction

Type 2 diabetes mellitus (T2DM) has become a serious public health problem (1), burdening health systems worldwide (2). An estimated 783 million people are expected to be affected by 2045 (2). T2DM is associated with a significant financial burden on patients and health systems alike (3). In addition, T2DM is strongly associated with the risk of macrovascular complications (4), and associated complications with a higher rate of mortality compared to patients with cardiovascular disease without diabetes. Together, these factors reduce patients' quality of life and underscore the urgency of developing effective management strategies.

Amid the escalating global prevalence of T2DM, effective prevention and control are essential, and Hemoglobin A1c (HbA1c) and fasting blood glucose (FBG) have been widely accepted as evaluation indicators. HbA1c is included in the American Diabetes Association diagnostic criteria for diabetes (5, 6), and effectively predicts diabetes complications (7). Fasting glucose is a core component of most T2DM risk assessment models (5) and is associated with increased risk of T2DM incidence (8). Both HbA1c and FPG serve as valuable screening tools for T2DM. Both HBA1c and FPG are valuable screening tools. Tight glycemic control can lower diabetes-related mortality by about 42% and decreases the risk of complications, thereby reducing costs (9, 10). Physical activity can delay the onset of T2DM or potentially prevent it through improvements in glycemic control (11, 12).

According to the World Health Organization, digital health refers to the applied practice of utilizing digital technologies, mobile technologies, and wireless technologies to support the achievement of health objectives. Compared with conventional care, digital health interventions (DHIs) help break distance and time barriers, making it widely accessible and reduce medical costs (13), Consequently, they are widely used in enhancing public health (14). DHIs have become essential tools for improving health outcomes in chronic disease self-management (15). They significantly enhance patients' health behavior patterns (16) and dietary behaviors (17, 18), thereby effectively supporting symptom control.

DHIs show promise for addressing physical inactivity due to their broad accessibility, precise population targeting, and cost-effective implementation (19), and the number of related journal publications continues to increase annually (20). Current evidence demonstrates that DHIs effectively promote physical activity in adult populations (21, 22). Yet several meta-analyses found these effects were not statistically significant (23, 24). This inconsistency likely stems from substantial methodological heterogeneity across studies, which obscures true effect magnitudes.

This systematic review and meta-analysis focused on the effects of DHIs on physical activity and glycemic control in patients with T2DM. Furthermore, we refined the classification of DHI groups to explore their impact on blood glucose control and physical activity promotion in T2DM patients in greater detail.

### 1.1 Methods

This systematic review and meta-analysis were prospectively registered in the PROSPERO international register (CRD420251032375) and conducted in accordance with the Preferred Reporting Items for Systematic Reviews and Meta-Analyses (PRISMA) guidelines (25). A completed PRISMA checklist is provided in Supplementary Appendix 1.

### 1.2 Search strategy

Two independent investigators (XHY and LY) systematically searched five electronic databases (Web of Science, Embase, Scopus, Cochrane Library, and PubMed) from inception through February 17, 2025. The retrieval-related terms are detailed in Supplementary Appendix 2 (Table S1).

### 1.3 Eligibility criteria

Studies were included if they met the following criteria: (1) participants diagnosed with T2DM; (2) study design was RCT; (3) intervention group received DHIs while control group received either no intervention, usual care, or conventional treatment; (4) the DHIs were specifically designed to influence physical activity and glycemic control in T2DM patients; and (5) primary or secondary outcomes included validated measures of physical activity and glycemic control.

Studies were excluded if they: (1) had incomplete data or were not published in English; (2) utilized digital technologies solely for data collection without intervention components, or implemented DHIs only at discrete timepoints (e.g., baseline and endpoint); or (3) combined DHIs with other concurrent intervention modalities in the experimental group, preventing isolation of DHI effects.

### 1.4 Study selection and data extraction

**Figure 1:**
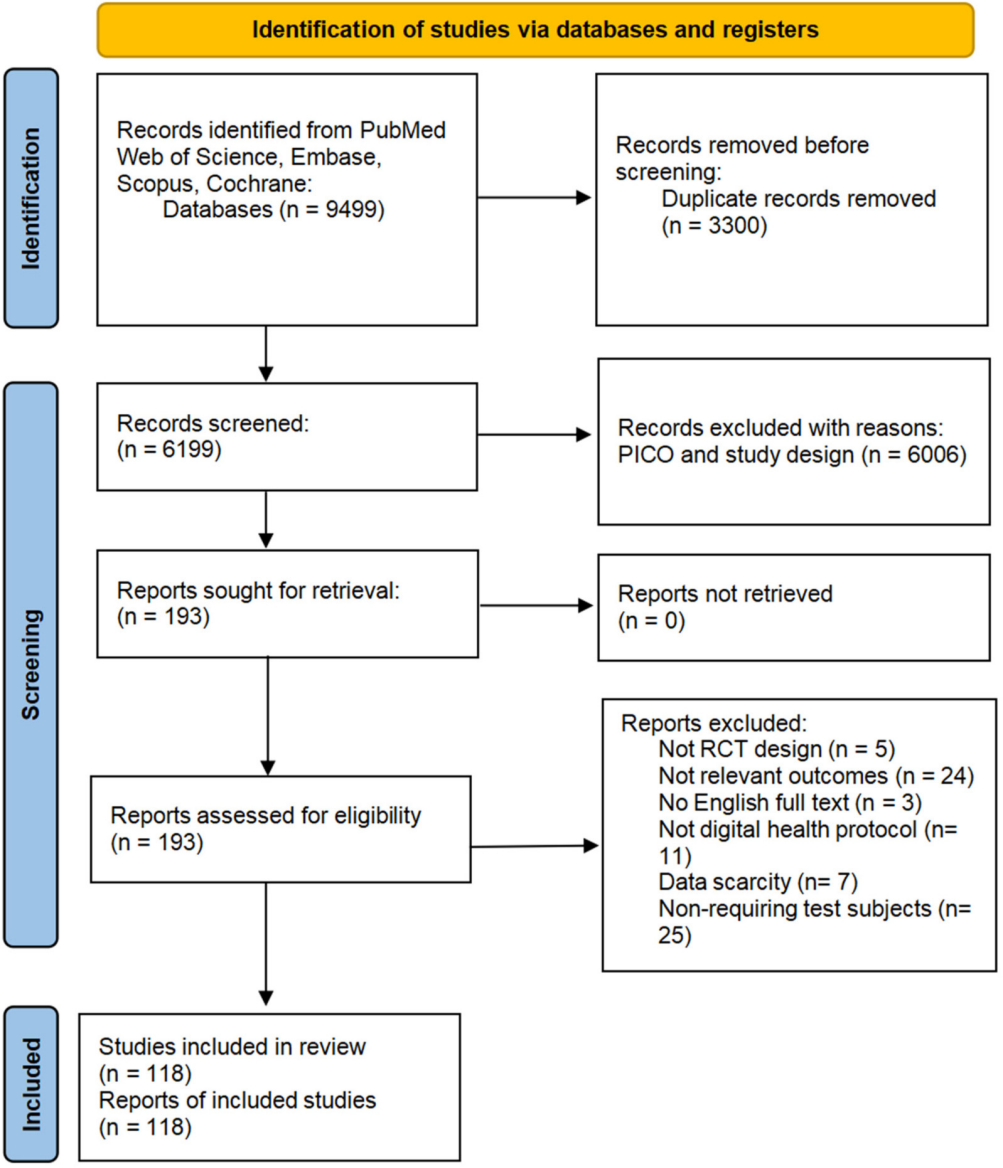
PRISMA flow diagram.

Following the importation of all records into EndNote X8 (Clarivate Analytics), title and abstract screening was systematically conducted to exclude irrelevant studies. The remaining articles underwent full-text evaluation against predefined inclusion criteria. For studies with inaccessible full texts or incomplete data, we contacted the author to obtain the necessary materials (Figure 1). Data extraction was performed using a standardized template that included: (1) intervention characteristics, including modality, duration; (2) participant demographics, such as diagnostic criteria, age distribution, and sample size; (3) glycemic outcomes, such as, HbA1c, FBG, Homeostatic Model Assessment of Insulin Resistance (HOMA-IR), and postprandial blood glucose (PBG); (4) physical activity metrics, such as, step counts and International Physical Activity Questionnaire (IPAQ) scores; (5) cost data (cost per person per month, standardized to US dollars). The literature screening and data extraction process was conducted by two independent investigators (LY and ZCR). Any discrepancies were resolved through discussion with a third researcher (XHY).

### 1.5 Data analysis

Data processing was performed using Review Manager 5.3. To address baseline heterogeneity observed in pre-intervention measurements of some included studies, we utilized the mean differences (MD) between post-intervention and baseline values for continuous outcomes. For studies employing heterogeneous measurement methodologies, standardized mean differences (SMD) were calculated.

The first step involved calculating the difference in means:

(1) $$M_{\mathrm{change}}=M_{\mathrm{post}}-M_{\mathrm{pre}}$$

Where *M*_change_ is the raw mean difference, *M*_post_ is the reported mean post-intervention, and *M*_pre_ is the reported mean preintervention.

Then the SD of the change in means is calculated as follows:

(2) $$SD_{\mathrm{change}}=\;\sqrt{{\mathrm{SD}}_{\mathrm{pre}}^{2}+\;{\mathrm{SD}}_{\mathrm{post}}^{2}-(2\times r\times SD_{\mathrm{pre}}\times SD_{\mathrm{post}})}$$

Among these variables, SD_change_ is the SD of the difference in means, SD_pre_ is the SD from pre-intervention, SD_post_ is the SD from post-intervention, and r is the pre-post test correlation coefficient. Correlation coefficients for pre- and post-intervention were rarely reported in the included studies. We therefore assumed *r* = 0.50, as recommended in the Cochrane Handbook.

Data transformation was systematically conducted according to the following protocol: For studies reporting outcomes as standard error (SE) or 95% confidence intervals (95% CI), conversions to standard deviations (SD) were performed using the embedded calculator in Review Manager. Missing change-from-baseline data were obtained through calculations using formulae from The Cochrane Handbook for Systematic Reviews of Interventions.

Heterogeneity was evaluated using Cochran's *Q* test and the *I*^2^ statistic. The magnitude of heterogeneity was interpreted by *I*^2^ value ranges: <25% (very low), 25%–50% (low), 50%–75% (moderate), and >75% (high) (26). Fixed-effect models were applied for low heterogeneity (*I*^2^ < 50%). Random-effects models were implemented for substantial heterogeneity (*I*^2^ ≥ 50%). Digital interventions in the study were categorized into four groups: mobile application group, phone call or SMS group, online platform group, and remote monitoring group. For indicators with insufficient included studies, we pooled them to evaluate their effect sizes, and subgroup analyses were additionally performed to assess heterogeneity. We conducted subgroup analyses of HbA1c levels for the telephone or SMS, online platform, and remote monitoring intervention groups, stratified by intervention duration when a sufficient number of studies were available (≥10 studies per subgroup).

### 1.6 Risk of bias

Two independent investigators (ZH and SYR) assessed the risk of bias and evidence quality of the included studies using the Cochrane Risk of Bias 2 (RoB 2) tool and the GRADE framework. Any discrepancies in the analyses were adjudicated by a third investigator (XHY) to ensure consensus. The funnel plot was used to detect the publication bias.

### 1.7 Sensitivity analysis

Sensitivity analyses were performed for outcomes with substantial heterogeneity (*I*^2^ > 50%), employing leave-one-out methodology. This approach involved iteratively removing individual studies demonstrating high heterogeneity or outlying effect sizes to assess their impact on the pooled estimates, evaluate the robustness of results, and identify potential sources of heterogeneity in the meta-analysis.

### 1.8 Meta-regression analysis

Random-effects meta-regression analyses were conducted using Stata 16.0 to quantitatively assess the potential influence of four prespecified covariates-research year, duration, intervention frequency, and sample size-on substantial heterogeneity.

## 2 Results

### 2.1 Search Results and study characteristics

The systematic search identified 9,499 potentially relevant records, with 118 studies meeting our criteria, including a total of 21,662 adults with T2DM. Sample sizes ranged from 19 to 1,012 participants across individual studies. Glycemic outcomes were heterogeneously reported, with HbA1c measured in 114 studies, FBG in 41 studies, PBG in 12 studies, and HOMA-IR in 4 studies. Additionally, 11 studies reported physical activity outcomes, 9 studies reported cost outcomes. The included studies were published between 2004 and 2024, with detailed characteristics of the included studies comprehensively summarized in Supplementary Appendix 2 (Table S3).

### 2.2 Risk of bias

Random sequence generation was high-risk in 14 studies, and allocation design was not concealed in 39. The implementation of researcher blinding was precluded in 42 studies owing to inherent methodological challenges associated with DHIs (27). Additionally, 40 studies exhibited high dropout rates (Supplementary Appendix 2, Figure S1). High certainty evidence demonstrated that the phone call or SMS and online platform interventions improve FBG. Moderate-certainty evidence supported phone calls or SMS and mobile application interventions for HbA1c reduction, as well as remote monitoring for FBG reduction. (Supplementary Appendix 2, Table S4). The details of publication bias are presented in Supplementary Appendix 2 (Figures S2–S6).

#### 2.3 Meta-analysis

##### 2.3.1 Hemoglobin A1c

**Figure 2:**
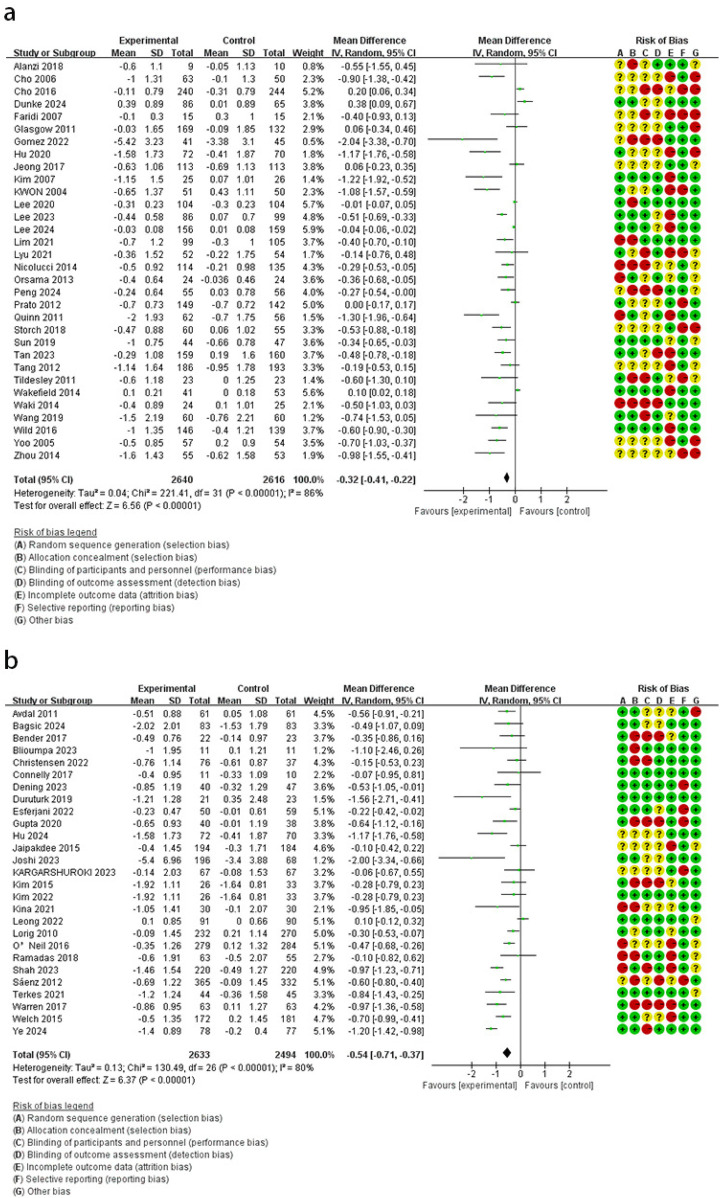
The effect of remote monitoring **(a)** and online platform **(b)** on HbA1c.

**Figure 3:**
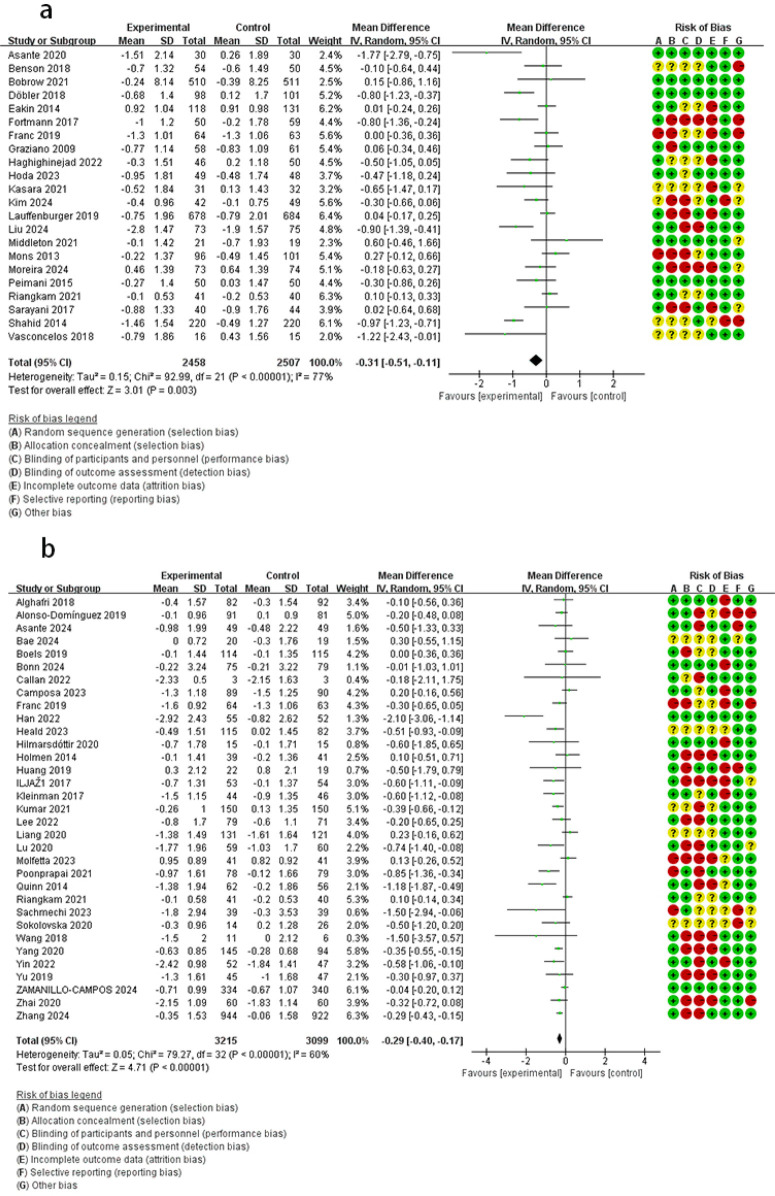
The effect of phone calls or SMS **(a)** and mobile application **(b)** on HbA1c.

Compared with the control group, the online platform intervention resulted in lower HbA1c levels (MD = −0.54, 95% CI: −0.71 to −0.37, *I*^2^ = 80%). Comparable effects were observed across interventions: remote monitoring (MD = −0.32, 95% CI: −0.41 to −0.22, *I*^2^ = 86%), mobile applications (MD = −0.29, 95% CI: −0.40 to −0.17, *I*^2^ = 69%), and phone calls or SMS (MD = −0.31, 95% CI: −0.51 to −0.11, *I*^2^ = 77%) (Figures 2,3).

##### 2.3.2 Fasting blood glucose

**Figure 4:**
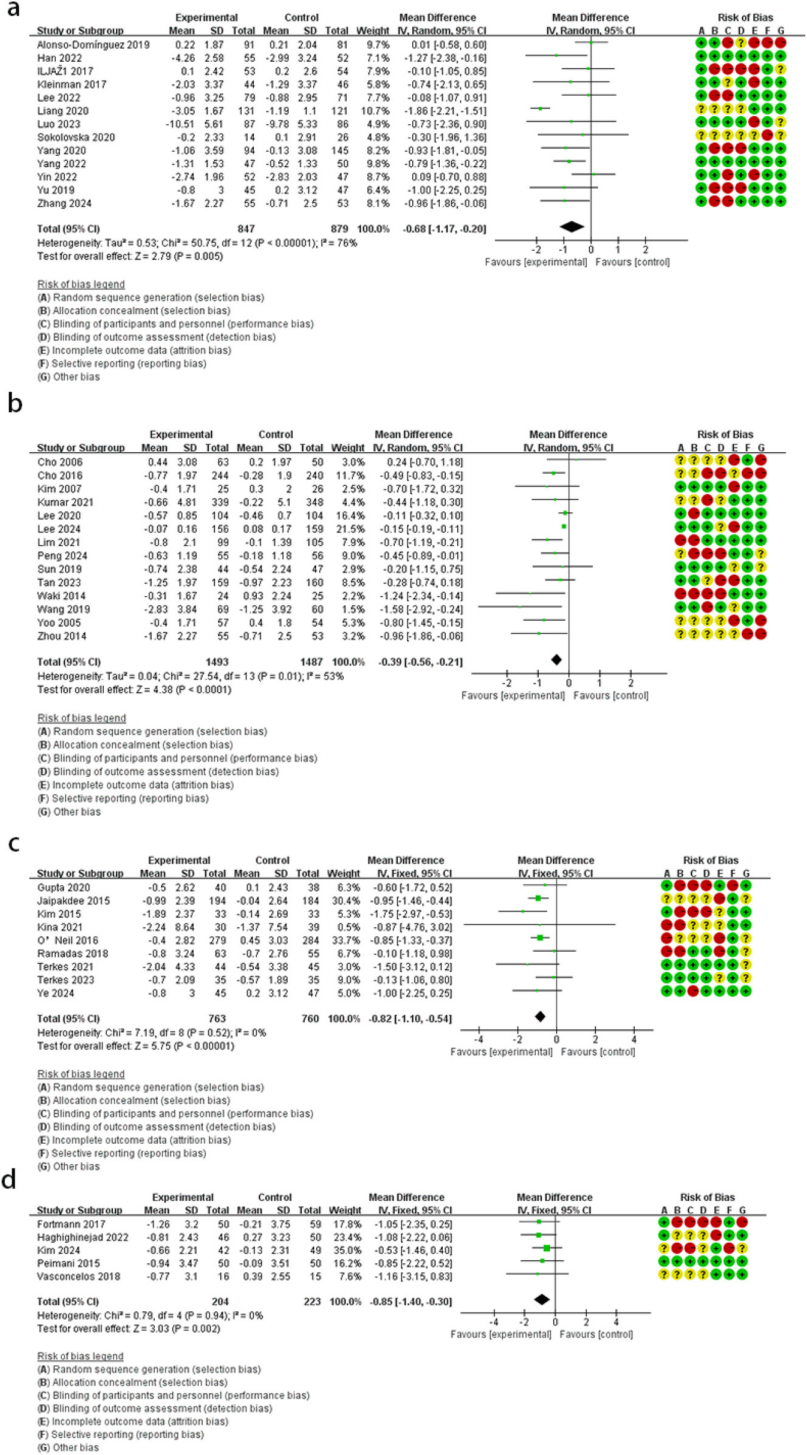
The effect of DHIs on FBG. **(a)** Mobile application; **(b)** remote monitoring; **(c)** online platform; **(d)** phone call or SMS.

The results across intervention groups demonstrated: compared with the control group, phone call or SMS group had lower FBG levels (MD = −0.85, 95% CI: −1.40 to −0.30, *I*^2^ = 0%); online platform group showed FBG lowering (MD = −0.82, 95% CI: −1.10 to −0.54, *I*^2^ = 0%); mobile application group exhibited clinically meaningful improvement (MD = −0.68, 95% CI: −1.17 to −0.20, *I*^2^ = 76%); remote monitoring group effectively controlled FBG (MD = −0.39, 95% CI: −0.56 to −0.21, *I*^2^ = 53%) (Figure 4).

##### 2.3.3 HOMA-IR

**Figure 5:**
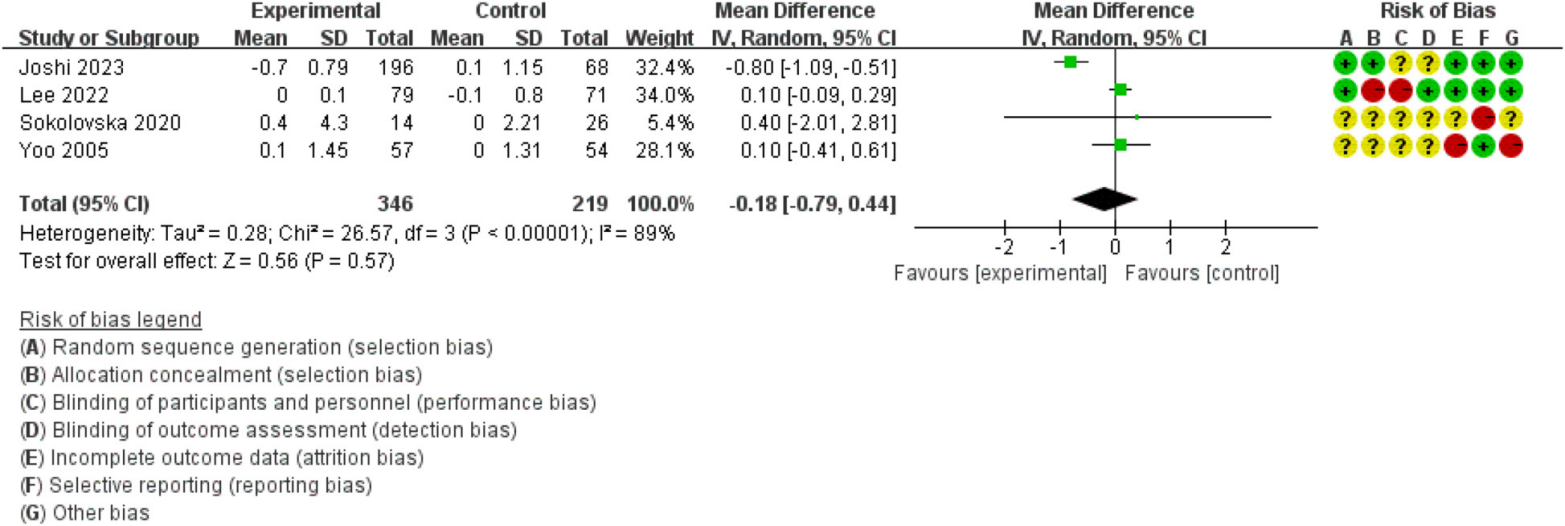
The effect of DHIs on HOMA-IR.

When compared to the control group, DHIs showed no improvement in HOMA-IR (MD = −0.18, 95% CI: −0.79 to 0.44, *I*^2^ = 89%) (Figure 5). Due to the limited number of included studies, only the overall effect size was evaluated, and no subgroup analyses were conducted.

##### 2.3.4 Postprandial blood glucose

**Figure 6:**
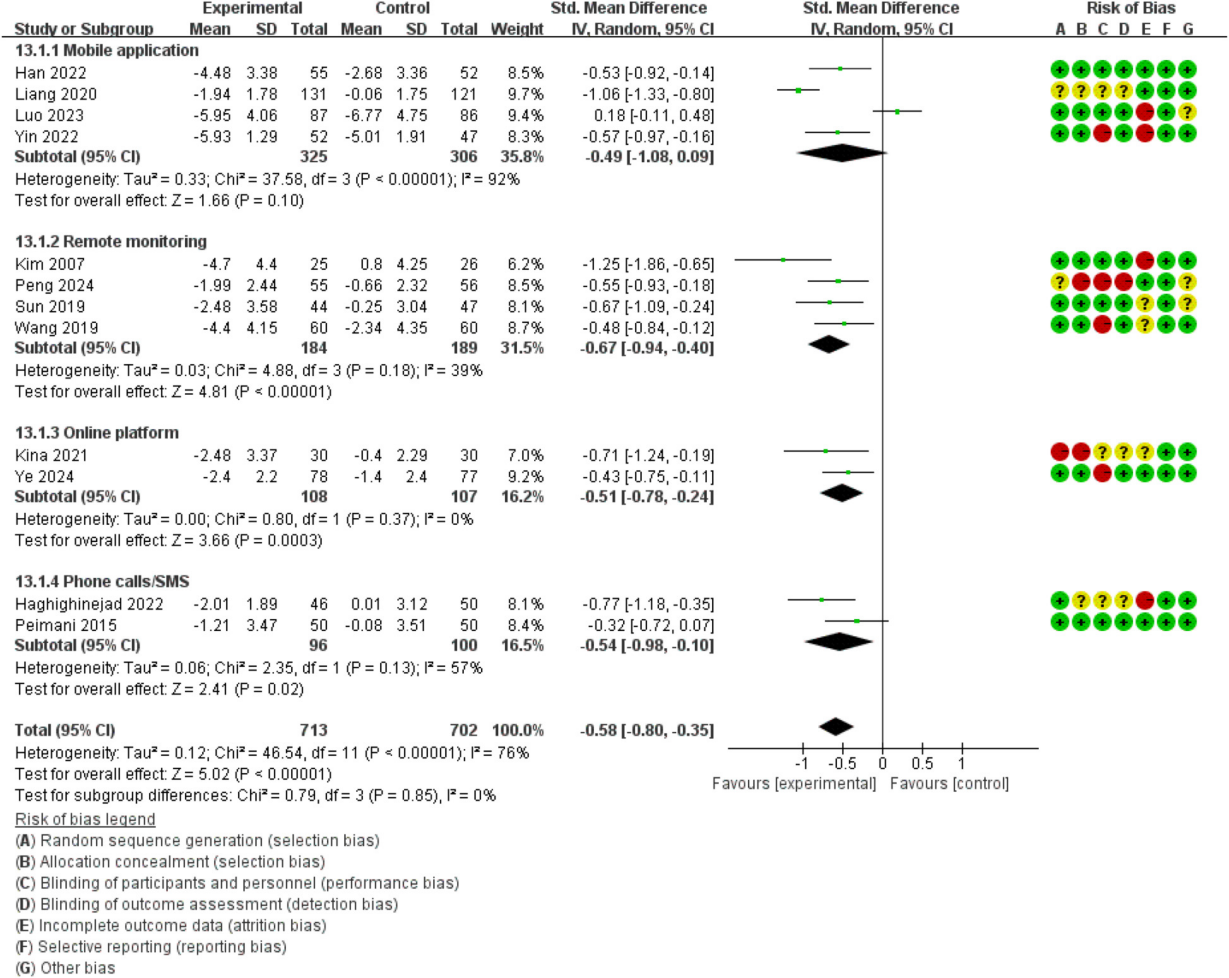
The effect of DHIs on PBG.

Compared with the control group, the forest plot (Figure 6) revealed that the intervention group had lower PBG levels (SMD = −0.58, 95% CI: −0.80 to −0.35, *I*^2^ = 76%).

Test of subgroup difference demonstrated no differences for PBG (*p* = 0.85, *I*^2^ = 0%). The mobile application group did not demonstrate a reduction in PBG levels by subgroup analyses (SMD = −0.49, 95% CI: −1.08 to 0.09, *I*^2^ = 92%). However, reductions were achieved in remote monitoring (SMD = −0.67, 95% CI: −0.94 to −0.40, *I*^2^ = 39%), online platform (SMD = −0.51, 95% CI: −0.78 to −0.24, *I*^2^ = 0%), and phone calls or SMS (SMD = −0.54, 95% CI: −0.98 to −0.10, *I*^2^ = 57%).

##### 2.3.5 Physical activity

**Figure 7:**
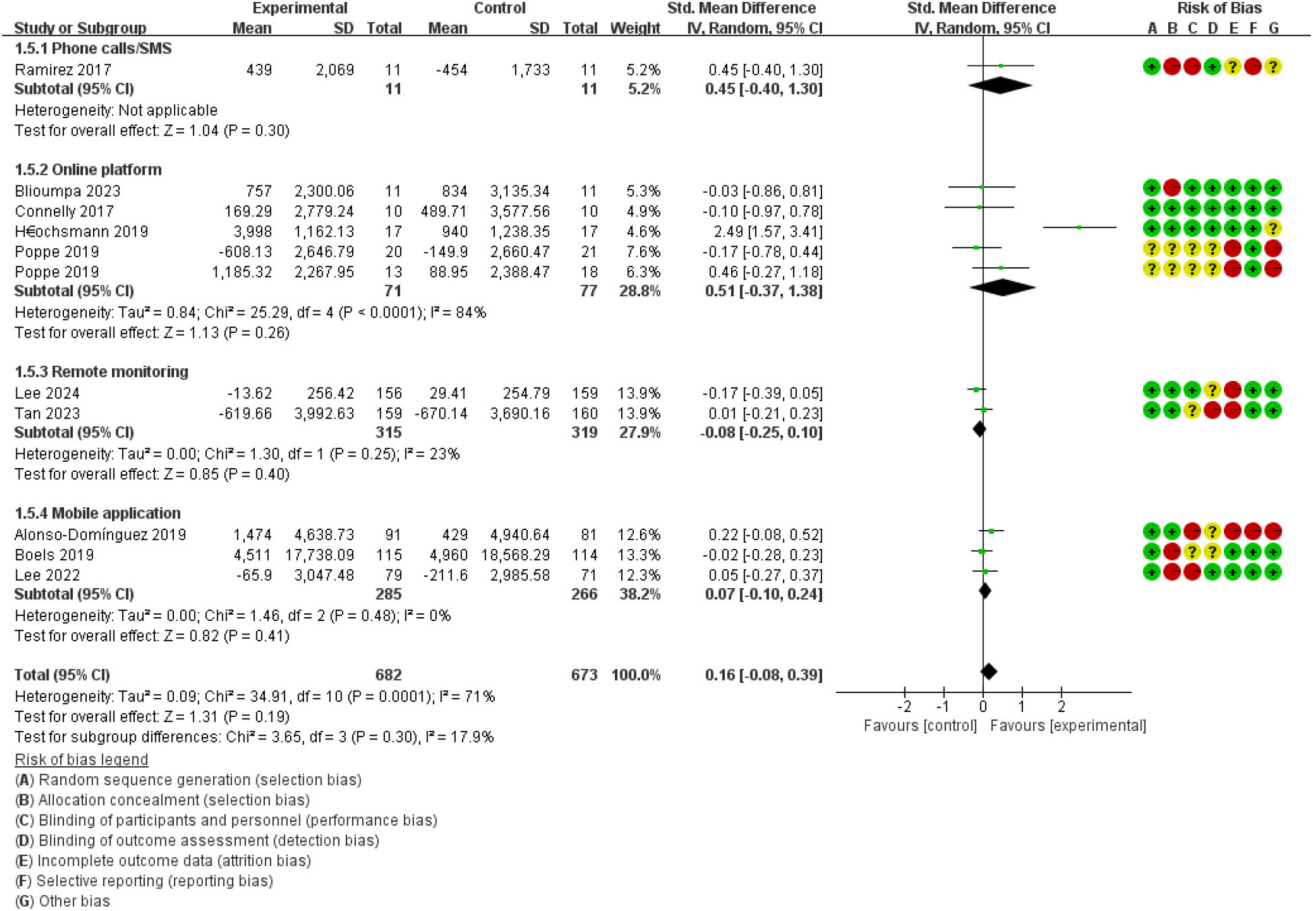
The effect of DHIs on physical activity.

Compared with the control group, the forest plot (Figure 7) revealed that DHIs could not promote physical activity in patients with T2DM (SMD = 0.16, 95% CI: −0.08 to 0.39, *I*^2^ = 71%).

In subgroup analyses, the test of subgroup difference demonstrated low heterogeneity for physical activity (*p* = 0.30, *I*^2^ = 17.9%). None of the DHIs could promote physical activity: online platform (SMD = 0.51, 95% CI: −0.37 to 1.3, *I*^2^ = 84%), remote monitoring (SMD = −0.08, 95% CI: −0.25 to 0.10, *I*^2^ = 23%), mobile application (SMD = 0.07, 95% CI: −0.10 to 0.24, *I*^2^ = 0%).

##### 2.3.6 Cost

Among the included studies, only nine reported economic data. Comparative cost analyses were conducted by evaluating mean values between intervention and control groups as reported in the included literature. Notably, among these comparative analyses, just one cost analysis showed that the experimental group incurred higher costs than the control group. When analyzing all included studies, the overall cost indicated that the experimental group (mean = $269.31) had lower costs than the control group (mean = $465.37) (Supplementary Appendix 2, Table S7).

### 2.4 Sensitivity analysis

For intervention groups exhibiting substantial heterogeneity, we excluded studies contributing to high heterogeneity in HbA1c levels within the online platform, telephone or SMS, and remote monitoring intervention groups, as well as in FBG and PBG levels within the mobile application intervention group. After removing Liang et al, heterogeneity fell sharply (*I*^2^ reduced from 76% to 8%) (MD = −0.3, 95% CI: −0.39 to −0.21), no significant changes were observed in other intervention groups. For other intervention groups, significant heterogeneity continues to be observed (Supplementary Appendix 2, Table S5).

### 2.5 Subgroup analysis

To identify consistent sources within the highly heterogeneous synthesized results, we conducted further subgroup analyses. PBG in the mobile app group was excluded from subgroup analysis due to insufficient studies. Continent contributed to heterogeneity in the telephone or SMS and online platform groups, while intervention duration may explain heterogeneity observed in the remote monitoring group, no significant subgroup differences were observed in any other analytical outcomes (Supplementary Appendix 2, Table S6).

### 2.6 Meta-regression

Regression analyses identified distinct moderator variables influencing HbA1c outcomes across intervention modalities: In the remote monitoring group, sample size (*p* = 0.008) may substantially influence our findings and could contribute to heterogeneity. Apart from this, no significant associations were detected between HbA1c results and the other measured indicators (Supplementary Appendix 2, Table S8).

## 3 Discussion

This meta-analysis demonstrated that, compared to the control group, DHIs significantly improved HbA1c, FBG, and PBG levels in patients with T2DM. However, no statistically significant improvements were observed in HOMA-IR or physical activity levels. Further subgroup analysis revealed that while three other DHIs modalities positively affected PBG, mobile application interventions failed to effectively control PBG levels in these patients. Additionally, none of the four intervention modalities demonstrated statistically significant improvements in physical activity compared to the control group.

Overall, DHIs showed positive effects on glycemic control in T2DM patients, consistent with prior studies (28). Compared to traditional treatments, DHIs offer greater portability, real-time feedback (29, 30), and continuous objective data recording (31). Smartphone ubiquity makes DHIs valuable in resource-limited or geographically remote settings (32). Beyond routine blood glucose monitoring, smartphone technologies facilitate systematic patient-provider communication (33), track daily meals, and calculate glycemic levels to recommend insulin dosages. Relevant information is uploaded in real-time via digital technology, potentially reducing time and economic costs for both patients and healthcare workers (34). In some cases, age and education level might influence the effectiveness of DHIs (35, 36). For instance, older adults demonstrate lower willingness to engage with DHIs due to factors such as health awareness and limited proficiency in using DHIs (37). However, targeted training can mitigate these barriers and improve overall outcomes (38).

However, discrepancies also exist between this study and earlier research. For example, some studies concluded that web-based interventions do not effectively improve blood glucose in T2DM patients (39), in contrast to our findings. This inconsistency may arise from differences in study design and inclusion criteria: prior meta-analyses excluded remote consultation and monitoring functionalities and focused only on T2DM patients with hypertension (comprising only 4 studies spanning 2009–2022). In contrast, our analysis included 27 studies (literature search extending to 2025) involving a broader T2DM population, thus enhancing external validity and generalizability. Regarding insulin sensitivity, while HOMA-IR remained statistically unchanged following intervention, evidence suggests reductions in leptin/adiponectin ratio (40), a marker typically associated with improved insulin sensitivity (41). This may indicate that improvements in endothelial function may not be primarily mediated by significant changes in body composition or biochemical parameters. Potential mechanistic explanations for the unchanged HOMA-IR could include enhanced hepatic insulin clearance (42) and compensatory reductions in insulin secretion, potentially secondary to improved peripheral glucose utilization (43). Collectively, the observed improvements in insulin sensitivity markers like the leptin/adiponectin ratio, despite stable HOMA-IR, could suggest that DHIs may affect insulin resistance via physiological pathways not fully reflected by conventional metrics.

Regarding physical activity, although some studies reported transient elevations in physical activity levels during DHI implementation (44), most investigations failed to demonstrate sustained improvements after intervention cessation (45), consistent with our findings. This pattern of initial responsiveness followed by regression to baseline activity levels likely reflects diminishing adherence to digitally-promoted exercise protocols over extended periods (46, 47). This may also be attributed to technical challenges and behavioral factors. Research has found that only 25% of participants maintained wearable tracking devices (e.g., pedometers) for at least 75% of the monitoring duration (48). Technical difficulties and failure to wear monitoring devices were identified as major barriers to optimal adherence in multiple studies, which compromises data integrity in technology-based interventions. To address these challenges, integrating behavioral change theories into DHIs can optimize digital tool design to enhance patients' physical activity. For instance, incorporating gamification elements such as point systems into DHIs increases patient engagement, promoting sustained participation in physical activity (49).

A primary advantage of digital health interventions lies in their substantial cost-effectiveness (50). Whereas conventional pharmaceutical development and deployment typically require hundreds of millions of US dollars (51), implementing digital health technologies incurs significantly lower costs. Furthermore, meta-analyses on T2DM indicate that despite substantial cost variations in DHIs due to technology types and device combinations, these interventions tend to demonstrate high cost-effectiveness (52). The cost-effectiveness data indicated that the intervention group generally performed better than the control group in most studies. Although a few studies reported higher costs in the intervention group, this was mainly due to post-study follow-up expenses and the additional costs of web-based glucose monitors and test strips. Overall, digital health technologies demonstrated better economic efficiency compared to conventional treatment.

Future studies should focus on optimizing DHIs by incorporating adaptive technologies that tailor interventions to individual patient profiles, such as baseline glycemic levels, comorbidities, and behavioral preferences. Investigating the integration of real-time biometric data with feedback could enhance personalization and long-term engagement. Rigorous, large-scale randomized controlled trials with extended follow-up periods (> 12 months) are needed to assess the durability of glycemic benefits and physical activity promotion. Additionally, exploring the synergistic effects of DHIs combined with clinician-led support or community-based programs may address current limitations in physical activity outcomes. Standardized reporting frameworks for DHI components (e.g., frequency, interactivity) are essential to reduce heterogeneity and enable cross-study comparisons. Third, further research is warranted to elucidate the specific physiological pathways through which DHIs may influence insulin sensitivity, extending beyond conventional metrics such as the HOMA-IR. Finally, efforts to ensure equitable access to DHIs, including linguistically and culturally adapted tools, will broaden their applicability and impact in global management of T2DM.

## 4 Limitation

This meta-analysis has several limitations that warrant consideration. First, the inclusion of exclusively English-language articles may introduce language bias, potentially omitting relevant studies from non-English-speaking regions. Future studies should incorporate multilingual literature to enhance the generalizability of findings. Second, the statistical power for specific outcomes (e.g., HOMA-IR and physical activity) was constrained by the limited number of included studies, potentially obscuring true intervention effects. Critically, a substantial proportion of trials exhibited high risk of bias or inadequate allocation concealment, further reducing confidence in the synthesized evidence. Finally, the long-term sustainability of glycemic improvements remains uncertain due to predominantly short-term follow-up periods (≤12 months) in most studies. Addressing these gaps requires standardized reporting frameworks and extended evaluations to assess effect durability.

## 5 Conclusion

This systematic review and meta-analysis demonstrate that DHIs significantly improve glycemic control in patients with T2DM, with consistent reductions in HbA1c and FBG across multiple modalities, including phone calls or SMS, mobile applications, online platforms, and remote monitoring. These findings highlight DHIs as scalable, cost-effective tools for integrating into routine diabetes care, particularly in resource-limited settings. However, DHIs showed no significant impact on enhancing physical activity or improving HOMA-IR. The heterogeneity in intervention designs and short follow-up durations limits conclusions on long-term efficacy. Future research should prioritize adaptive DHIs that personalize feedback and address barriers to physical activity adherence while evaluating sustainability beyond 12 months.

## Data Availability

The original contributions presented in the study are included in the article/Supplementary Material, further inquiries can be directed to the corresponding author.
