# Supplementary material for: The effectiveness of digital health intervention on glycemic control and physical activity in patients with type 2 diabetes: a systematic review and meta-analysis
Source: Front Digit Health. 2025 Jul 29;7:1630588. doi: 10.3389/fdgth.2025.1630588 (PMC12340998; doi:10.3389/fdgth.2025.1630588)
Supplement: Supplementary file 1 [file Datasheet1.pdf]

Supplementary Table 1 Search string for each database

Subject term	Search term
Participant	Type 2 diabetes mellitus OR Type 2 DM OR T2DM OR Non-insulin dependent diabetes OR NIDDM OR Diabetes mellitus type 2 OR Type 2 diabetes OR Diabetes type 2
Intervention	Smartphone OR Phone OR Mobile OR Mhealth OR M-health OR Ehealth OR E-health OR Tablet OR Telemedicine OR Telehealth OR Digital health OR Email OR E-mail OR Web-based OR Online OR App OR Application OR Internet OR Software OR Message OR SMS OR Computers OR Media OR Video game OR Online game OR Virtual reality
Outcome	Exercis* OR Sport* OR "Physical activit*" OR Fitness OR PA OR "Motor activit*" OR "Step count" OR Walk*<br>Haemoglobin A1c OR Hemoglobin A1c OR Glycated hemoglobin OR Glycated haemoglobin OR HbA1c OR Blood sugar OR Blood glucose OR Fasting glucose OR Insulin resistance OR HOMA-IR OR HOMA IR OR Homeostasis model assessment OR Glucose tolerance test OR OGTT OR Glucose intolerance OR Glycemic OR Glycaemia OR Glycaemic OR Glycemia OR A1c
Research design	"Randomized controlled trial"

Supplementary Table 2 Include the article information summary

Category		Article quantity
Intervention type	Remote monitoring	33
	Mobile application	35
	Online platform	30
	Phone calls or SMS	23
Outcome indicator	HbA1c	114
	FBG	41
	PBG	12
	HOMA-IR	4
	PA	11
Duration	≤ 3 months	28
	3 months − 9 months	47
	> 9 months	41
	NR	2
Sample	0 − 150	75
	150 − 300	25
	> 300	18

FBG: Fasting blood glucose; HbA1c: Glycated hemoglobin; PA: Physical activity; PBG: Postprandial blood glucose; NR: Not report

Supplementary Table 3 The basic information of the included article (continued)

Type	Study	Subject	Age	Sample (n, control)	Intervention	Duration	Outcome
Remote monitoring	Alanzi 2018 (1)	T2DM	≤50	19 (10)	Remote monitoring	6 months	HbA1c
	Cho 2006 (2)	T2DM	NR	113 (50)	Remote monitoring, phone calls or SMS	30 months	HbA1c<br>FBG
	Cho 2016 (3)	T2DM	NR	484 (240)	Remote monitoring	6 months	HbA1c<br>FBG
	Dunkel 2024 (4)	T2DM	40-67	151（65）	Remote monitoring, phone calls or SMS	12 months	HbA1c
	Faridi 2008 (5)	T2DM	≥18	30 (15)	Remote monitoring, phone calls or SMS	3 months	HbA1c
	Glasgow 2012 (6)	T2DM, heart disease	25-75	463 (132)	Remote monitoring	12 months	HbA1c
	Gomez 2022 (7)	T2DM	NR	86 (45)	Remote monitoring, phone calls or SMS	3 months	HbA1c
	Hu 2021 (8)	T2DM	≥18	142 (70)	Remote monitoring	24 months	HbA1c
	Jeong 2018 (9)	T2DM	NR	338 (113)	Remote monitoring, phone calls or SMS	24 weeks	HbA1c
	Kim 2007 (10)	T2DM	NR	51 (26)	Phone calls or SMS, remote monitoring	12 months	HbA1c<br>FBG<br>2h-PBG
	Kumar 2021 (11)	T2DM	NR	687 (348)	Remote monitoring, phone calls or SMS	12 months	FBG
	KWON 2004 (12)	T2DM	≥30	101 (50)	Remote monitoring	12 months	HbA1c
	Lee 2020 (13)	T2DM	18-75	240 (120)	Remote monitoring	52weeks	HbA1c<br>FBG
	Lee 2023 (14)	T2DM	19-69	274 (99)	Remote monitoring, phone calls or SMS	48 weeks	HbA1c
	Lee 2024 (15)	T2DM	26-65	315 (159)	Remote monitoring, mobile application	12 months	HbA1c<br>FBG
	Lim 2021 (16)	T2DM, BMI ≥	22-72	204 (105)	Mobile application, remote monitoring	6 months	HbA1c

Supplementary Table 3 The basic information of the included article (continued)

Type	Study	Subject	Age	Sample (n, control)	Intervention	Duration	Outcome
		23					FBG
	Lyu 2021 (17)	T2DM	NR	106 （52）	Remote monitoring	3 months	HbA1c
	Nicolucci 2015 (18)	T2DM	≥45	249 (135)	Remote monitoring, phone calls or SMS	12 months	HbA1c
	Orsama 2013 (19)	T2DM	30-70	48 (24)	Remote monitoring, mobile application	10 months	HbA1c
	Peng 2024 (20)	T2DM	18-70	111 (56)	Remote monitoring	3 months	HbA1c<br>FBG<br>2h-PBG
	Prato 2012 (21)	T2DM	NR	291 (142)	Remote monitoring	24 weeks	HbA1c
	Quinn 2011 (22)	T2DM	18-64	79 (56)	Remote monitoring, mobile application, phone calls or SMS	12 months	HbA1c
	Storch 2019 (23)	T2DM	40-67	115 (55)	Remote monitoring	12 months	HbA1c
	Sun 2019 (24)	T2DM	≥65	91 (47)	Remote monitoring, mobile application, phone calls or SMS	3 months	HbA1c<br>FBG<br>PBG
	Tan 2023 (25)	T2DM	26-65	319 (160)	Remote monitoring, mobile application	6 months	HbA1c<br>FBG<br>PA (IPAQ)
	Tang 2013 (26)	T2DM	≥18	397 (193)	Remote monitoring, phone calls or SMS	12 months	HbA1c
	Tildesley 2011 (27)	T2DM	NR	46 (23)	Remote monitoring	12 months	HbA1c
	Wakefield 2014 (28)	T2DM and hypertension	NR	94 (53)	Remote monitoring	12 weeks	HbA1c
	Waki 2014 (29)	T2DM	NR	49(25)	Phone calls or SMS, remote monitoring	3 months	HbA1c<br>FBG

Supplementary Table 3 The basic information of the included article (continued)

Type	Study	Subject	Age	Sample (n, control)	Intervention	Duration	Outcome
Mobile application	Wang 2019 (30)	T2DM	30-60	353 (181)	Mobile application, remote monitoring	6 months	HbA1c<br>FBG<br>2h-PBG
	Wild 2016 (31)	T2DM	≥17	285 (139)	Remote monitoring	9 months	HbA1c
	Yoo 2009 (32)	T2DM and hypertension	30-70	111（54）	Remote monitoring	12 weeks	HbA1c<br>FBG<br>HOMA-IR
	Zhou 2014 (33)	T2DM	18-75	108 (53)	Remote monitoring, phone calls or SMS	3 months	HbA1c<br>FBG
	Alghafri 2018 (34)	T2DM	22-68	174 (92)	Mobile application	12 months	HbA1c
	Alonso-Domínguez 2019 (35)	T2DM	25-70	172 (81)	Mobile application	NR	HbA1c<br>FBG<br>PA (step count)<br>PA (IPAQ)
	Asante 2024 (36)	T2DM	NR	98 (49)	Mobile application	3 months	HbA1c
	Bae 2024 (37)	T2DM and depressive symptoms	20-80	39 (19)	Mobile application	12 weeks	HbA1c
	Boels 2019 (38)	T2DM	40-70	129 (115)	Mobile application, phone calls or SMS	9 months	HbA1c
	Bonn 2024 (39)	T2DM	NR	154 (79)	Mobile application	6 months	HbA1c
	Callan 2022 (40)	T2DM	NR	12 (3)	Mobile application	12 weeks	HbA1c
	Zamanillo-Campos 2023 (41)	T2DM	≥18	179 (90)	Mobile application	3 months	HbA1c

Supplementary Table 3 The basic information of the included article (continued)

Type	Study	Subject	Age	Sample (n, control)	Intervention	Duration	Outcome
	Franc 2019 (42)	T2DM	NR	191 (63)	Mobile application, phone calls or SMS	13 months	HbA1c
	Han 2022 (43)	T2DM	NR	107 (52)	Mobile application	12 months	HbA1c<br>FBG<br>2h-PBG
	Heald 2023 (44)	T2DM	NR	197 (82)	Mobile application	6 months	HbA1c
	Hilmarsdóttir 2020 (45)	T2DM	NR	30 (15)	Mobile application	6 months	HbA1c
	Holmen 2014 (46)	T2DM	NR	120 (41)	Mobile application	12 months	HbA1c
	Huang 2019 (47)	T2DM	NR	41 (19)	Mobile application	12 weeks	HbA1c
	ILJAŽ1 2017 (48)	T2DM	NR	107 (54)	Mobile application	12 months	HbA1c
	Kleinman 2017 (49)	T2DM	18-65	90 (46)	Mobile application	6 months	HbA1c<br>FBG
	Kumar 2021 (11)	T2DM	18-65	300 (150)	Mobile application	6 months	HbA1c
	Lee 2022 (50)	T2DM	19-74	234 (71)	mobile application, phone calls or SMS	26 weeks	HbA1c<br>FBG<br>HOMA-IR<br>PA (step count)
	Liang 2020 (51)	T2DM	20-70	252 (121)	Mobile application	6 months	HbA1c<br>FBG<br>2h-PBG
	Lu 2021 (52)	T2DM	NR	119 (60)	Mobile application	NR	HbA1c
	Luo 2023 (53)	T2DM	NR	173 (86)	Mobile application	12 months	FBG<br>2h-PBG
	Di Molfetta 2023 (54)	T2DM	NR	82 (41)	Mobile application	16 weeks	HbA1c

Supplementary Table 3　The basic information of the included article (continued)

Type	Study	Subject	Age	Sample (n, control)	Intervention	Duration	Outcome
	Poonprapai 2022 (55)	T2DM	≥65	157 (79)	Mobile application	9 months	HbA1c
	Quinn 2016 (56)	T2DM	18-64	118 (56)	Mobile application, phone calls or SMS	12 months	HbA1c
	Riangkam 2021 (57)	T2MD	18-60	122 (40)	Mobile application, phone calls or SMS	3 months	HbA1c
	Sachmechi 2023 (58)	T2DM	18	78 (39)	Mobile application	12 weeks	HbA1c
	Sokolovska 2020 (59)	T2DM	35-75	40 (26)	Mobile application	4 months	HbA1c<br>FBG<br>HOMA-IR
	Wang 2018 (60)	T2DM	21-75	17 (6)	Mobile application	6 months	HbA1c
	Yang 2020 (61)	T2DM	≥18	239 (94)	Mobile application	3 months	HbA1c<br>FBG
	Yang 2022 (62)	T2DM	40-60	97 (50)	Mobile application, phone calls or SMS	12 months	HbA1c<br>FBG
	Yin 2022 (63)	T2DM	NR	99 (47)	Phone calls or SMS, mobile application	6 months	HbA1c<br>FBG<br>PBG
	Yu 2019 (64)	T2DM	35-65	92 （47）	Mobile application	24 weeks	HbA1c<br>FBG
	Zamanill-campos 2024 (65)	T2DM	≥18	674 (340)	Mobile application	12 months	HbA1c
	Zhai 2020 (66)	T2DM	18-69	120 (60）	Mobile application	6 months	HbA1c
	Zhang 2024 (67)	T2DM	≥40	1866 (922)	Mobile application	24 months	HbA1c<br>FBG
	Avdal 2011(68)	T2DM	≥18	122 (61)	Online platform	6 months	HbA1c

Supplementary Table 3 The basic information of the included article (continued)

Type	Study	Subject	Age	Sample (n, control)	Intervention	Duration	Outcome
Online platform	Spierling Bagsic 2023 (69)	T2DM	≥18	166 (83)	Online platform	6 months	HbA1c
	Bender 2017 (70)	T2DM	NR	45 (23)	Online platform	3 months	HbA1c
	Blioumpa 2023 (71)	T2DM	NR	22 (11)	Online platform	6 weeks	HbA1c<br>PA (IPAQ)
	Christensen 2022 (72)	T2DM	NR	128 (53)	Online platform	6 months	HbA1c
	Connelly 2017 (73)	T2DM	NR	31 (10)	Online platform	6 months	HbA1c<br>PA (step count)<br>PA (ActiGraph)
	Dening 2023 (74)	T2DM	40-89	87 （47）	Online platform	16 weeks	HbA1c
	Duruturk 2019 (75)	T2DM	NR	50 (25)	Online platform	6 weeks	HbA1c
	Esferjani 2022 (76)	T2DM	≥60	118 (59)	Mobile application, online platform	3 months	HbA1c
	Gong 2020 (77)	T2DM	NR	145 (79)	Online platform	12 months	HbA1c
	Gupta 2020 (78)	T2DM	NR	78 (40)	Mobile application, online platform	4 months	HbA1c<br>FBG
	H€ochsmann 2019 (79)	T2DM	NR	35 (17)	Online platform	24 weeks	HbA1c<br>PA (step count)
	Jaipakdee 2015 (80)	T2DM	NR	378 (184)	Online platform	6 months	HbA1c<br>FBG
	Welch 2015 (81)	T2DM	≥18	353 (181)	Online platform	6 months	HbA1c
	Joshi 2023 (82)	T2DM	18-70	264 (68)	Online platform, phone calls or SMS	12 months	HbA1c<br>HOMA-IR
	Kargarshuroki 2023 (83)	T2DM	NR	134(67)	Online platform	45 days	HbA1c
	Kim 2022 (84)	T2DM	≥19	59 (33)	Online platform	6 months	HbA1c

Supplementary Table 3 The basic information of the included article (continued)

Type	Study	Subject	Age	Sample (n, control)	Intervention	Duration	Outcome
	Kim 2015 (85)	T2DM	60-85	66 (33)	Online platform, phone calls or SMS	6 months	HbA1c<br>FBG
	Kina 2022 (86)	T2DM	20-65	60 (30)	Phone calls or SMS, mobile application, online platform	6 months	HbA1c<br>FBG<br>2h-PBG
	Leong 2022 (87)	T2DM	≥18	181 (90)	Online platform	3 months	HbA1c
	Lorig 2010 (88)	T2DM	NR	645 (238)	Online platform	18 months	HbA1c
	O'Neil 2016 (89)	T2DM	18-70	563 (284)	Online platform, phone calls or SMS	12 months	HbA1c<br>FBG
	Poppe 2019 (90)	T2DM	≥18	41 (18)	Online platform, mobile application	12 months	PA (IPAQ)<br>PA (Accelerometer)<br>PA (step count)
		T2DM	≥18	55 (21)	Online platform, mobile application	12 months	PA (IPAQ)<br>PA (Accelerometer)<br>PA (step count)
	Ramadas 2018 (91)	T2DM	NR	118 (55)	Online platform	6 months	HbA1c<br>FBG
	Shah 2024 (92)	T2DM	18-85	183 (91)	Online platform	6 months	HbA1c
	Sáenz 2012 (93)	T2DM	NR	697 (332)	Mobile application, online platform	18 months	HbA1c
	Terkes 2024 (94)	T2DM	18-65	89 (45)	Online platform, phone calls or SMS	3 months	HbA1c<br>FBG
	Terkes 2023 (95)	T2DM	≥65	70 (35)	Mobile application, online platform	6 weeks	FBG
	Warren 2017 (96)	T2DM	≥18	126 (63)	Online platform	12 months	HbA1c

Supplementary Table 3 The basic information of the included article (continued)

Type	Study	Subject	Age	Sample (n, control)	Intervention	Duration	Outcome
	Ye 2024 (97)	T2DM and hypertensive complications	NR	155 (77)	Phone calls or SMS, mobile application, online platform	26 weeks	HbA1c<br>FBG<br>2h-PBG
	Asante 2020 (98)	T2DM	NR	60 (30)	Phone calls or SMS	12 months	HbA1c
	Benson 2019 (99)	T2DM	40-75	104 (50)	Phone calls or SMS	12 months	HbA1c
	Farmer 2021 (100)	T2DM	NR	1012 (510）	Phone calls or SMS	12 months	HbA1c
	Döbler 2018 (101)	T2DM	NR	199 (101)	Phone calls or SMS	12 months	HbA1c
	Eakin 2014 (102)	T2DM	NR	249 (131)	Phone calls or SMS	18 months	HbA1c<br>PA (Accelerometer)
	Fortmann 2017 (103)	T2DM	18-75	109 (59)	Phone calls or SMS	6 months	HbA1c<br>FBG
	Franc 2019 (42)	T2DM	NR	191 (63)	Phone calls or SMS	13 months	HbA1c
	Graziano 2009 (104)	T2DM	NR	119 （58）	Phone calls or SMS	3 months	HbA1c
	Haghighinejad 2022 (105)	T2DM	NR	96 (50)	Phone calls or SMS	3 months	HbA1c<br>FBG<br>2h-PBG
Phone calls or SMS	Hoda 2023 (106)	T2DM	NR	97 (48)	Phone calls or SMS	3 months	HbA1c
	Kasar 2022 (107)	T2DM	NR	63 (32)	Phone calls or SMS	12 weeks	HbA1c
	Kim 2024 (108)	T2DM	NR	91 (49)	Phone calls or SMS	12 weeks	HbA1c<br>FBG
	Lauffenburger 2019 (109)	T2DM	18-64	1362 (684)	Phone calls or SMS	12 months	HbA1c
	Liu 2024 (110)	T2DM	≥18	148 (75)	Phone calls or SMS	12 weeks	HbA1c

Supplementary Table 3 The basic information of the included article (continued)

Type	Study	Subject	Age	Sample (n, control)	Intervention	Duration	Outcome
	Middleton 2021 (111)	T2DM	18-40	40 (19)	Phone calls or SMS	12 months	HbA1c
	Mons 2013 (112)	T2DM	NR	197 (101)	Phone calls or SMS	12 months	HbA1c
	Moreira 2024 (113)	T2DM	NR	147 (74)	Phone calls or SMS	12 months	HbA1c
	Peimani 2016 (114)	T2DM	18-79	150 (50)	Phone calls or SMS	3 months	HbA1c<br>FBG<br>2h-PBG
	Riangkam 2022 (57)	T2DM	18-60	122 (40)	Phone calls or SMS	3 months	HbA1c
	Ramirez 2017 (115)	T2DM	≥18	22 (11)	Phone calls or SMS	12 weeks	PA (step count)
	Sarayani 2017 (116)	T2DM	NR	84 (44)	Phone calls or SMS	9 months	HbA1c
	Shahid 2015 (117)	T2DM	18-70	440 (220)	Phone calls or SMS	6 months	HbA1c
	Hérica Cristina Alves de Vasconcelos 2015 (118)	T2DM	NR	31 (15)	Phone calls or SMS	24 weeks	HbA1c<br>FBG

FBG: Fasting blood glucose; HbA1c: Glycated hemoglobin; 2h-PBG: 2 - hour Postprandial blood glucose; PA: Physical activity; PBG: Postprandial blood glucose; MVPA: Moderate-to-vigorous physical activity; NR: Not report

Supplementary Table 4 The results of GRADE's evaluation of each level of evidence

Indicator	Intervention	Sample		Risk of bias	Inconsistency	Indirectness	Imprecision	Other considerations	Certainty
		Experimental	Control						
HOMA-IR		346	219	serious	serious	not serious	not serious	publication bias strongly suspected	very low
Physical activity		681	674	serious	serious	serious	not serious	publication bias strongly suspected	very low
Fasting blood glucose	Mobile application	847	879	serious	not serious	not serious	not serious	publication bias strongly suspected	low
	Phone calls or SMS	204	223	serious	not serious	not serious	not serious	publication bias strongly suspected	high
	Remote monitoring	1493	1487	not serious	not serious	not serious	not serious	publication bias strongly suspected	moderate
	Online platform	763	760	serious	not serious	not serious	not serious	strong association	high
HbA1c	Phone calls or SMS	2458	2507	not serious	serious	not serious	not serious	none	moderate
	Mobile application	3215	3099	not serious	not serious	not serious	not serious	publication bias strongly suspected	moderate
	Remote monitoring	2640	2616	serious	serious	not serious	not serious	none	low
	Online platform	2633	2494	serious	serious	not serious	not serious	none	low
Postprandial Blood Glucos		713	702	not serious	not serious	not serious	not serious	none	high

Supplementary Table 5 Leave-one-out sensitivity analysis for high heterogeneity

Indicators	Intervention methods	Delete articles	Heterogeneity	MD (95% CI) or SMD (95% CI)	studies (n)
HbA1c	Phone calls or SMS	Vasconcelos 2018	78%	-0.29 [-0.49, -0.09]	21
		Middleton 2021	78%	-0.33 [-0.53, -0.13]	21
		Asante 2020	76%	-0.27 [-0.46, -0.07]	21
		Shahid 2014	65%	-0.24 [-0.42, -0.07]	21
		Farmer 2021	78%	-0.32 [-0.52, -0.12]	21
	Online platform	Joshi 2023	80%	-0.52 [-0.69, -0.36]	26
		Duruturk 2019	80%	-0.52 [-0.69, -0.36]	26
		Ye 2024	71%	-0.49 [-0.64, -0.35]	26
		Leong 2022	75%	-0.57 [-0.73, -0.41]	26
	Remote monitoring	Gomez 2022	86%	-0.31 [-0.40, -0.21]	31
		Hu 2020	86%	-0.30 [-0.39, -0.20]	31
		Wakefield 2014	86%	-0.35 [-0.45, -0.25]	31
		Dunke 2024	86%	-0.34 [-0.44, -0.25]	31
		Faridi 2007	86%	-0.31 [-0.41, -0.22]	31
		Kim 2007	86%	-0.30 [-0.39, -0.21]	31
FBG	Mobile application	Liang 2020	8%	-0.30 [-0.39, -0.21]	12
PBG	Mobile application	Liang 2020	84%	-0.29 [-0.81, 0.23]	3
		Luo 2023	71%	-0.74 [-1.12, -0.37]	3

Supplementary Table 6 Subgroup analysis for high heterogeneity

Intervention	Subgroup		No. of studies	MD	95% CI		p	I^2
Phone calls or SMS	Publication year	2005-2015	4	-0.37	-0.5	-0.23	<0.001	0.883
		2015-2025	18	-0.12	-0.19	-0.05	<0.001	0.7
	Continent	North America	3	0.02	-0.08	0.12	0.884	0
		Europe	5	-0.21	-0.36	-0.06	0.002	0.761
		Oceania	2	0.06	-0.17	0.29	0.312	0.02
		Asia	8	-0.42	-0.54	-0.3	0.003	0.677
		Africa	1	NA	NA	NA	NA	NA
		South America	2	-0.23	-0.52	0.07	0.154	0.508
	Duration	0-6	16	-0.16	-0.24	-0.08	<0.001	0.782
		6-12	13	-0.34	-0.42	-0.26	<0.001	0.817
		>12	3	-0.38	-0.54	-0.19	<0.001	0.817
Online platform	Publication year	2005-2015	6	-0.34	-0.42	-0.25	0.013	0.654
		2015-2025	20	-0.44	-0.51	-0.36	<0.001	0.794
	Continent	Europe	8	-0.46	-0.58	-0.34	0.609	0
		North America	3	-0.42	-0.59	-0.25	0.443	0
		Oceania	5	-0.44	-0.54	-0.35	0.001	0.774
		Asia	10	-0.27	-0.38	-0.17	<0.001	0.884
	Duration	0-6	21	-0.4	-0.48	-0.33	<0.001	0.798
		6-12	3	-0.42	-0.55	-0.29	0.039	0.7
		>12	2	-0.36	-0.47	-0.24	0.066	0.705
Remote monitoring	Publication year	2005-2015	14	-0.32	-0.42	-0.23	<0.001	0.793
		2015-2025	18	-0.23	-0.30	-0.16	<0.001	0.814
	Continent	Europe	7	-0.21	-0.33	-0.10	<0.001	0.793

Intervention		Subgroup	No. of studies	MD	95% CI		*p*	I^2
		Asia	19	-0.29	-0.36	-0.22	<0.001	0.814
		North America	5	-0.10	-0.27	0.06	<0.001	0.827
		South America	1	NA	NA	NA	NA	NA
		0-6	11	-0.42	-0.53	-0.31	0.005	0.605
	Duration	6-12	8	-0.06	-0.14	0.03	<0.001	0.781
		>12	2	0.01	-0.2	0.21	0.964	0

NA: Not Application

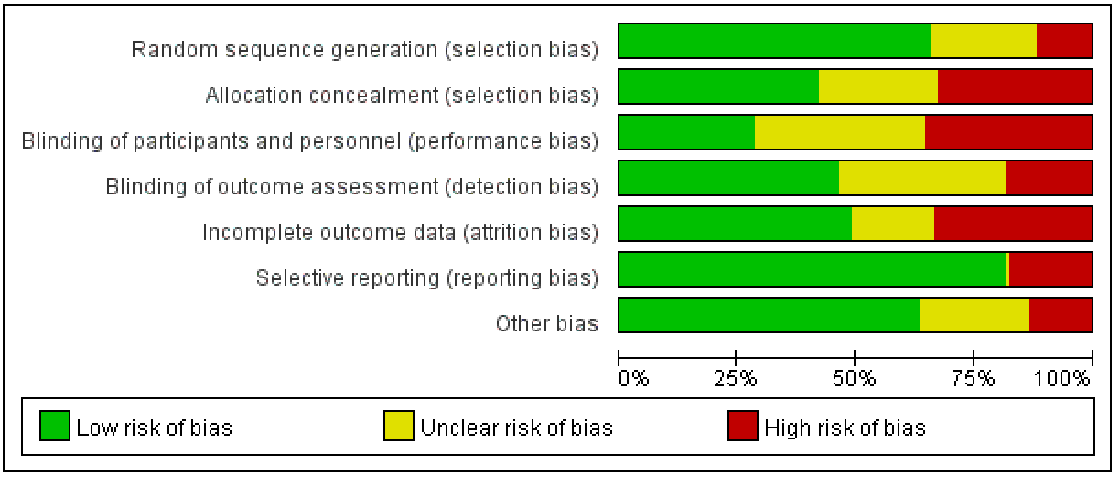

Supplementary Figure 1 The results of the Cochrane risk of bias assessment for the included studies.

Online platform:

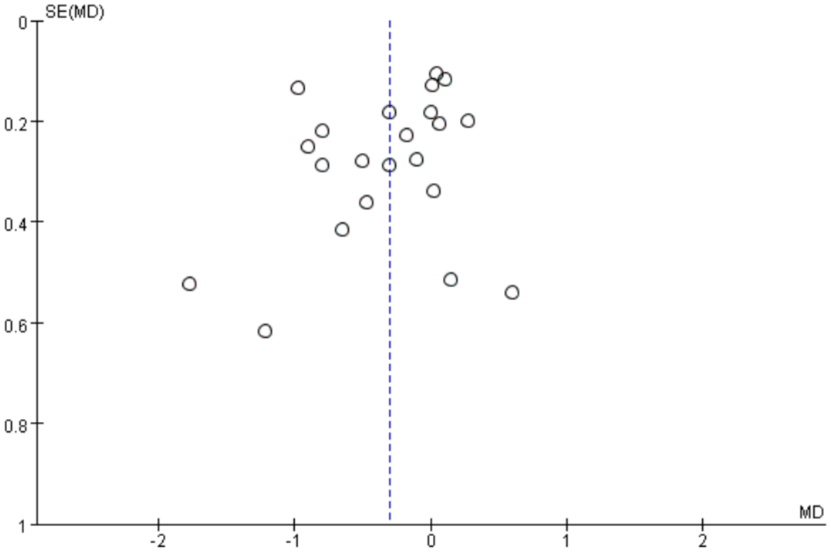

Remote monitoring:

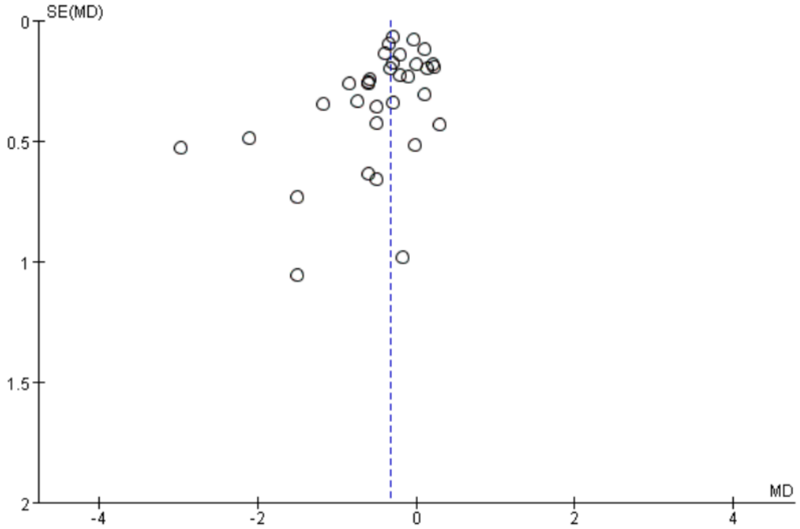

Supplementary Figure 2 Funnel plot of the effect of remote monitoring and online platforms on HbA1c

Mobile application:

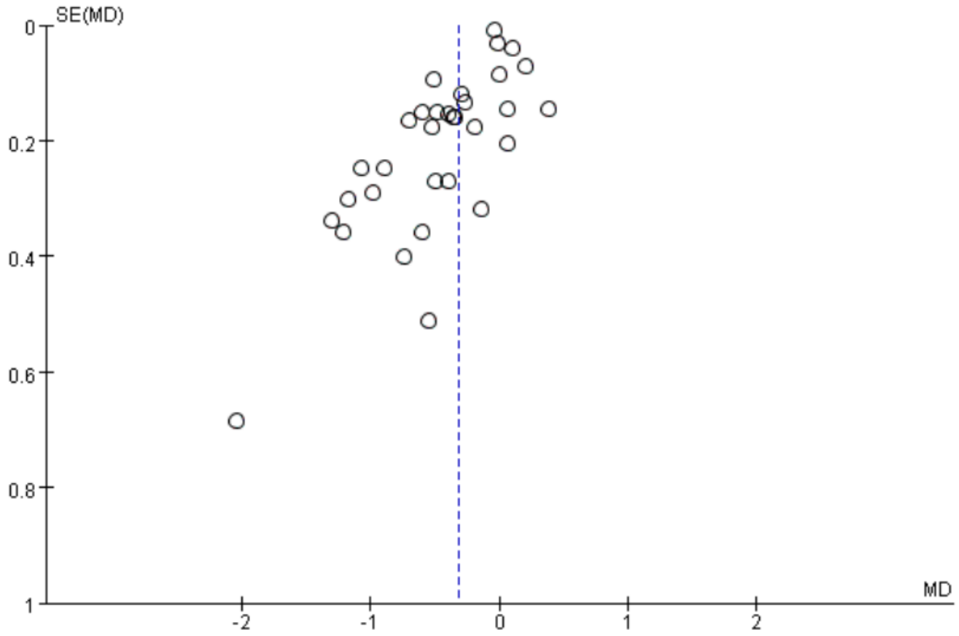

Phone calls or SMS:

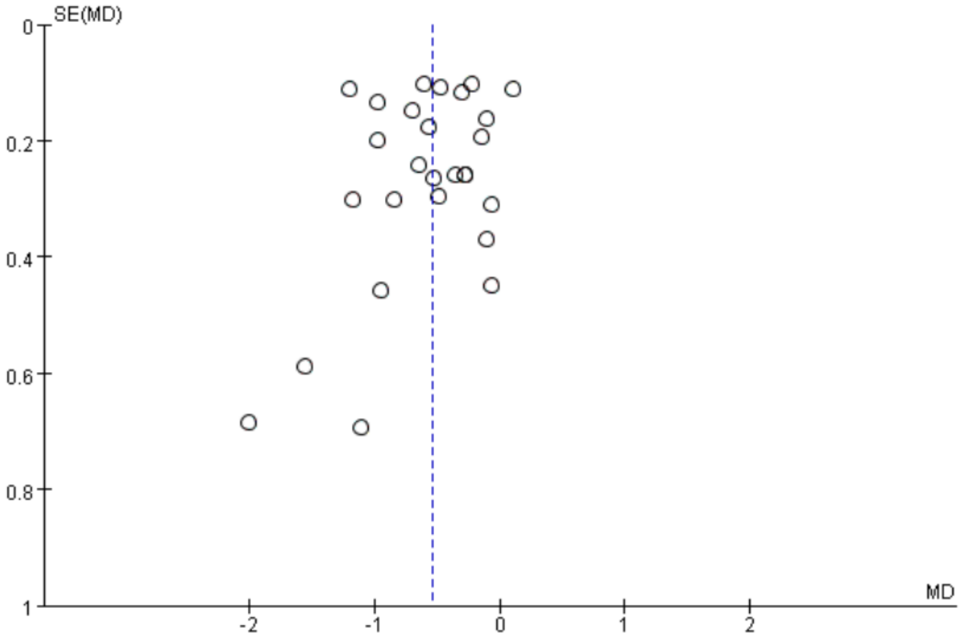

Supplementary Figure 3 Funnel plot of the effect of mobile application and phone calls or SMS on HbA1c

Phone calls or SMS:

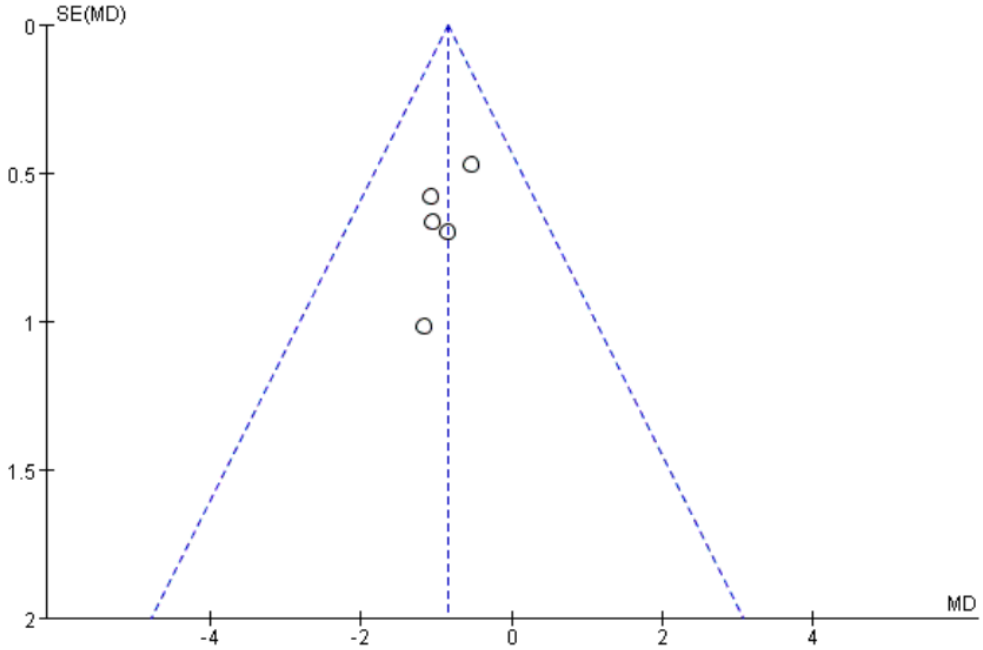

Mobile application:

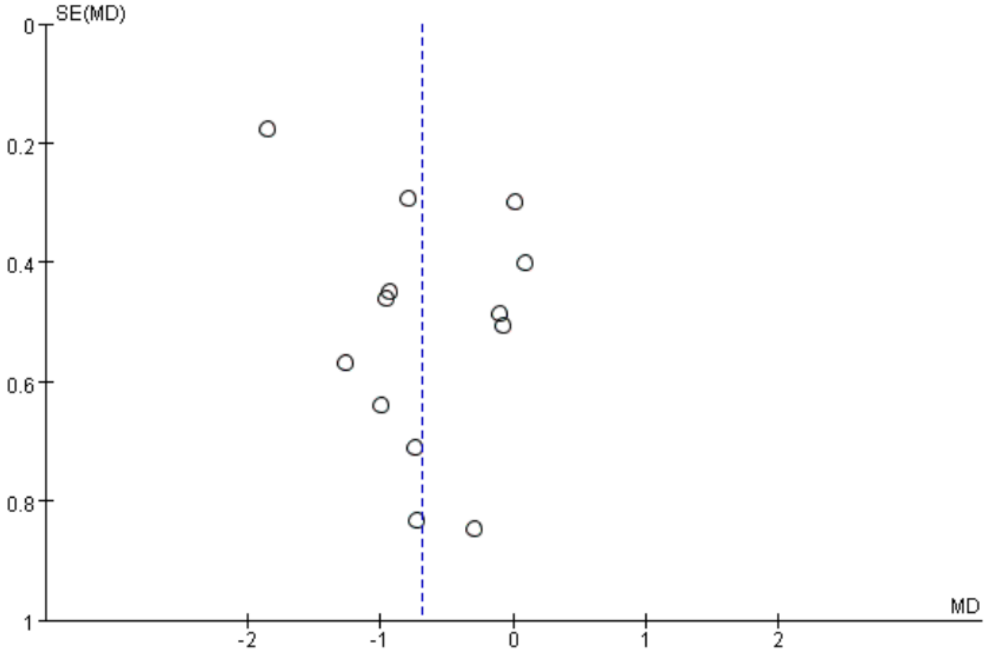

Supplementary Figure 4 Funnel plot of the effect of the phone calls or SMS and mobile application on FBG

Remote monitoring:

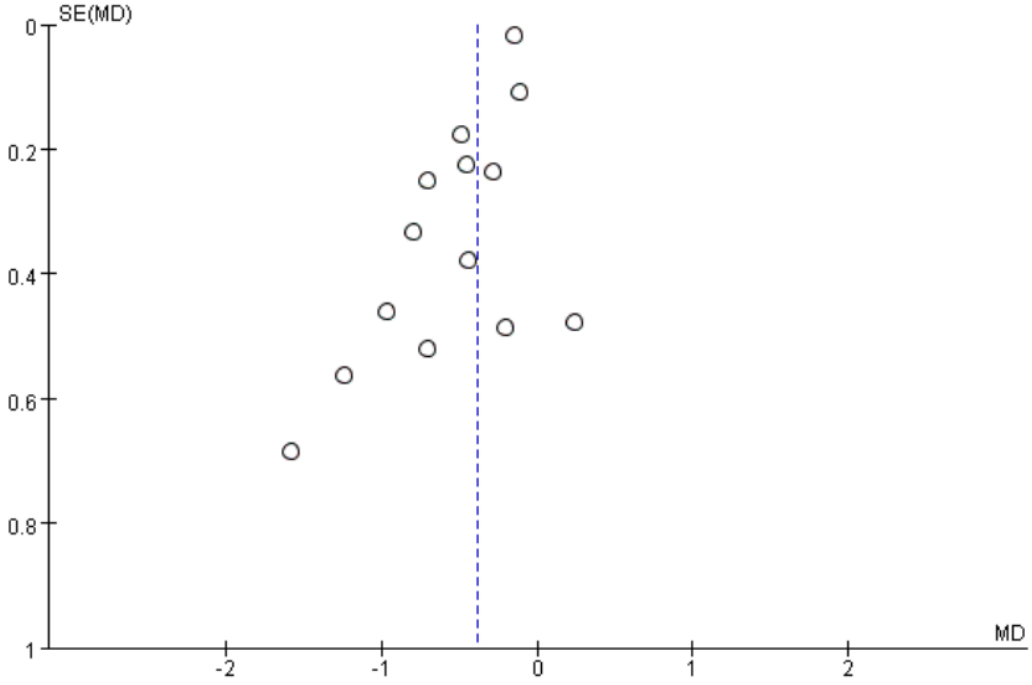

Online platform:

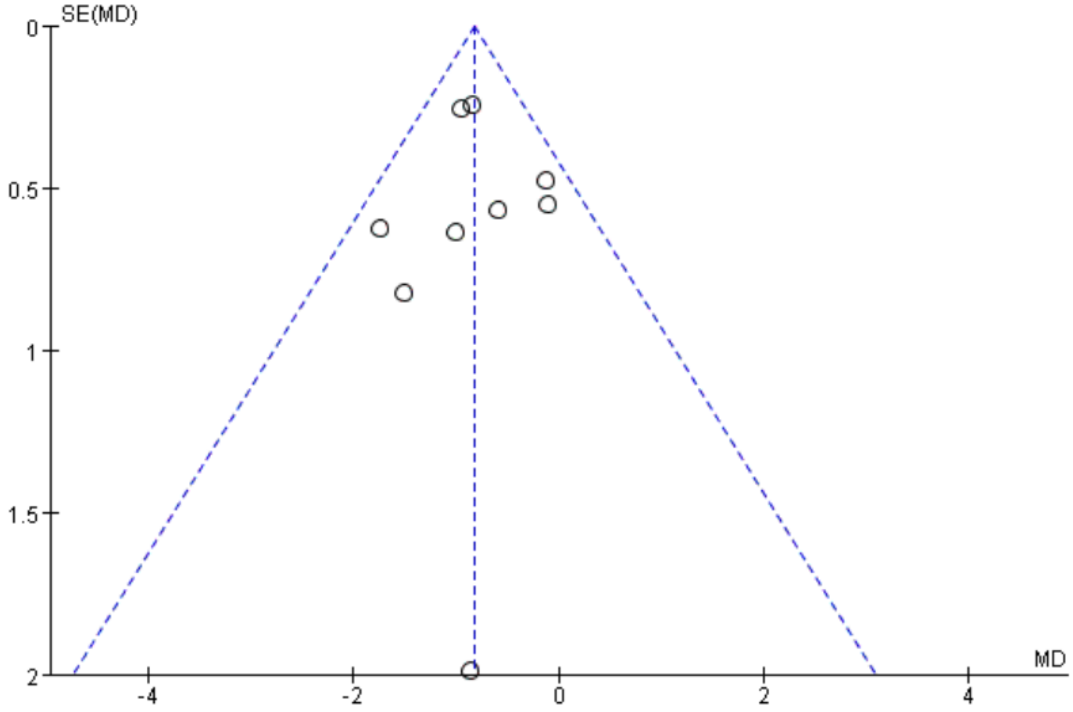

Supplementary Figure 5 Funnel plot of the effect of remote monitoring and online platform on FBG

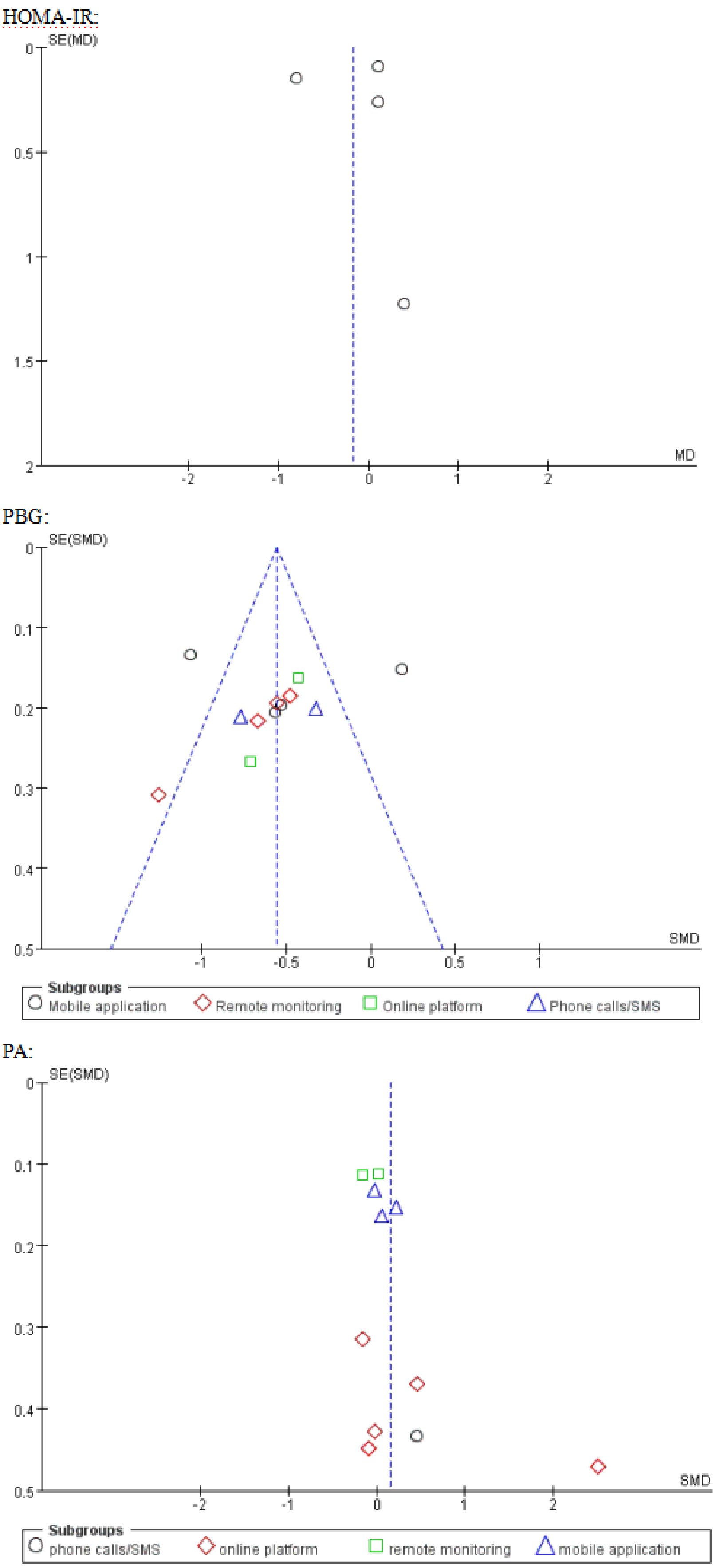

Supplementary Figure 6 Funnel plot of the effect of DHIs on HOMA-IR, PBG, and PA

Supplementary Table 7 The cost-effectiveness of including the article

Study	Cost per person-month (USD)		
	Experimental group	Control group	Difference
Asante (36)	571.8	678.3	-106.5
Farmer (100)	194.1	1757.4	-1563.3
Haghighinejad (105)	113.1	221.1	-108
Lee (13)	801	636	165
Lim (16)	127.8	153.6	-25.8
Lu (52)	126.6	130.2	-3.6
Warren (96)	428.4	528.36	-99.96
Yang (62)	27.09	46.43	-19.34
Zhang (67)	33.9	36.9	-3
Average	269.31	465.37	-196.06

Supplementary Table 8 Meta-regression results of HbA1c

Intervention	Value	No. of studies	p	β	95% CI	F
Phona calls or SMS	Year	21	0.506	-0.0127	-0.0519, 0.0265	0.02
	Frequency	20	0.818	0.0027	-0.0213, 0.0267	0.11
	Duration	21	0.184	0.0202	-0.0105, 0.0510	5.78
	Sample	21	0.565	0.0001	-0.0003, 0.0006	8.94
Online platform	Year	26	0.433	-0.8000	-0.0478, 0.0212	0.96
	Frequency	19	0.832	-0.0011	-0.0122, 0.0099	0.13
	Duration	26	0.946	-0.0011	-0.0348, 0.0326	0.00
	Sample	26	0.731	0.0001	-0.0007, 0.0009	0.02
Remote monitoring	Year	32	0.183	0.0163	-0.0081, 0.0409	0.03
	Frequency	21	0.293	0.0068	-0.064, 0.0200	0.41
	Duration	32	0.229	-0.0130	-0.0359, 0.0089	3.17
	Sample	32	0.008	0.0015	-0.0004, 0.0026	14.83

Reference

1. Alanzi T, Alanazi NR, Istepanian R, Philip N. Evaluation of the effectiveness of mobile diabetes management system with social networking and cognitive behavioural therapy (CBT) for T2D. mHealth. 2018;4:35.
2. Cho J, Chang S, Kwon H, Choi Y, Ko S, Moon S, et al. Long-term effect of the Internet-based glucose monitoring system on HbA1c reduction and glucose stability: a 30-month follow-up study for diabetes management with ubiquitous medical care system. Diabetes care. 2006;2006 Dec; 29(12):2625.
3. Cho JH, Kim HS, Yoo SH, Jung CH, Lee WJ, Park CY, et al. An Internet-based health gateway device for interactive communication and automatic data uploading: clinical efficacy for type 2 diabetes in a multi-centre trial. Journal of telemedicine and telecare. 2017;23(6):595-604.
4. Dunkel A, von Storch K, Hochheim M, Zank S, Polidori MC, Woopen C. Long-term effects of a telemedically-assisted lifestyle intervention on glycemic control in patients with type 2 diabetes – A two-armed randomised controlled trial in Germany. Journal of diabetes and metabolic disorders. 2024;23(1):519-32.
5. Faridi Z, Liberti L, Shuval K, Northrup V, Ali A, Katz DL. Evaluating the impact of mobile telephone technology on type 2 diabetic patients' self-management: the NICHE pilot study. Journal of evaluation in clinical practice. 2008;14(3):465-9.
6. Glasgow RE, Kurz D, King D, Dickman JM, Faber AJ, Halterman E, et al. Twelve-month outcomes of an Internet-based diabetes self-management support program. Patient education and counseling. 2012;87(1):81-92.
7. Maria Gomez A, Cristina Henao D, Leon Vargas F, Mauricio Munoz O, David Lucero O, Garcia Jaramillo M, et al. Efficacy of the mHealth application in patients with type 2 diabetes transitioning from inpatient to outpatient care: a randomized controlled clinical trial. Diabetes research and clinical practice. 2022;189:109948.
8. Hu Y, Wen X, Ni L, Wang F, Hu S, Fang F. Effects of telemedicine intervention on the management of diabetic complications in type 2 diabetes. International journal of diabetes in developing countries. 2021;41(2):322-8.
9. Jeong JY, Jeon JH, Bae KH, Choi YK, Park KG, Kim JG, et al. Smart Care Based on Telemonitoring and Telemedicine for Type 2 Diabetes Care: Multi-Center Randomized Controlled Trial. Telemedicine journal and e-health : the official journal of the American Telemedicine Association. 2018;24(8):604-13.
10. Kim HS. A randomized controlled trial of a nurse short-message service by cellular phone for people with diabetes. International journal of nursing studies. 2007;44(5):687-92.
11. Kumar D, Joshi A, Grover A, Raina S, Bhardwaj AK, Malhotra B, et al. Effect of personalized human-centered dietary decision support system (PHCDDSS) on dietary knowledge, attitude, practice (KAP), and mean fasting blood sugar (FBS) among participants with type 2 diabetes mellitus (T2DM) in community-based settings of northern state of India. Journal of Diabetology. 2021;12(3):338-43.
12. Kwon HS, Cho JH, Kim HS, Song BR, Ko SH, Lee JM, et al. Establishment of Blood Glucose Monitoring System Using the Internet. Diabetes care. 2004;27(2):478-83.
13. Lee JY, Chan CKY, Chua SS, Ng CJ, Paraidathathu T, Lee KKC, et al. Telemonitoring and Team-Based Management of Glycemic Control on People with Type 2 Diabetes: a Cluster-Randomized Controlled Trial. Journal of general internal medicine. 2020;35(1):87-94.
14. Lee YB, Kim G, Jun JE, Park H, Lee WJ, Hwang YC, et al. An Integrated Digital Health Care Platform for Diabetes Management With AI-Based Dietary Management: 48-Week Results From a Randomized Controlled Trial. Diabetes care. 2023;46(5):959-66.
15. Lee CS, Tyagi S, Ling Koh EY, Gong PP, Ang SB, Gerald Koh CH, et al. Health outcomes of telemonitoring of patients with type-2 diabetes mellitus: one-year results from a randomized controlled trial (Optimizing care of Patients via Telemedicine In Monitoring and aUgmenting their control of diabetes Mellitus). Journal of telemedicine and telecare. 2024:1357633X241261733.

16. Lim SL, Ong KW, Johal J, Han CY, Yap QV, Chan YH, et al. Effect of a Smartphone App on Weight Change and Metabolic Outcomes in Asian Adults With Type 2 Diabetes: a Randomized Clinical Trial. JAMA network open. 2021;4(6):e2112417.
17. Lyu QY, Huang JW, Li YX, Chen QL, Yu XX, Wang J, et al. Effects of a nurse led web-based transitional care program on the glycemic control and quality of life post hospital discharge in patients with type 2 diabetes: A randomized controlled trial. International journal of nursing studies. 2021;119:103929.
18. Nicolucci A, Cercone S, Chiriatti A, Muscas F, Gensini G, Grp RS. A Randomized Trial on Home Telemonitoring for the Management of Metabolic and Cardiovascular Risk in Patients with Type 2 Diabetes. Diabetes technology & therapeutics. 2015;17(8):563-70.
19. Orsama AL, Lähteenmäki J, Harno K, Kulju M, Wintergerst E, Schachner H, et al. Active assistance technology reduces glycosylated hemoglobin and weight in individuals with type 2 diabetes: Results of a theory-based randomized trial. Diabetes Technology and Therapeutics. 2013;15(8):662-9.
20. Peng P, Shen Y, Xiong H. Wearable monitoring device based on an internet management platform improves metabolic parameters in type 2 diabetes patients: a prospective pilot study. Postgraduate medicine. 2024;136(5):523-32.
21. Del Prato S, Nicolucci A, Lovagnini-Scher AC, Turco S, Leotta S, Vespasiani G, et al. Telecare Provides Comparable Efficacy to Conventional Self-Monitored Blood Glucose in Patients with Type 2 Diabetes Titrating One Injection of Insulin Glulisine-the ELEONOR Study. Diabetes technology & therapeutics. 2012;14(2):175-82.
22. Quinn CC, Shardell MD, Terrin ML, Barr EA, Ballew SH, Gruber-Baldini AL. Cluster-randomized trial of a mobile phone personalized behavioral intervention for blood glucose control. Diabetes care. 2011;34(9):1934-42.
23. von Storch K, Graaf E, Wunderlich M, Rietz C, Polidori MC, Woopen C. Telemedicine-Assisted Self-Management Program for Type 2 Diabetes Patients. Diabetes technology & therapeutics. 2019;21(9):514-21.
24. Sun C, Sun L, Xi S, Zhang H, Wang H, Feng Y, et al. Mobile phone–Based telemedicine practice in older chinese patients with type 2 diabetes mellitus: Randomized controlled trial. JMIR mHealth and uHealth. 2019;7(1).
25. Tan NC, Tyagi S, Lee CS, Koh EYL, Goh KLS, Gong PP, et al. Effectiveness of an algorithm-driven home telemonitoring system on the metabolic control and self-care behaviour of Asian adults with type-2 diabetes mellitus: a randomised controlled trial. Journal of telemedicine and telecare. 2023:1357633X231203490.
26. Tang PC, Overhage JM, Chan AS, Brown NL, Aghighi B, Entwistle MP, et al. Online disease management of diabetes: engaging and motivating patients online with enhanced resources-diabetes (EMPOWER-D), a randomized controlled trial. Journal of the American Medical Informatics Association : JAMIA. 2013;20(3):526-34.
27. Tildesley HD, Mazanderani AB, Chan JHM, Ross SA. Efficacy of A1C reduction using internet intervention in patients with type 2 diabetes treated with insulin. Canadian journal of diabetes. 2011;35(3):250-3.
28. Wakefield BJ, Koopman RJ, Keplinger LE, Bomar M, Bernt B, Johanning JL, et al. Effect of home telemonitoring on glycemic and blood pressure control in primary care clinic patients with diabetes. Telemedicine journal and e-health. 2014;20(3):199-205.
29. Waki K, Fujita H, Uchimura Y, Omae K, Aramaki E, Kato S, et al. DialBetics: a novel smartphone-based self-management support system for type 2 diabetes patients. Journal of diabetes science and technology. 2014;8(2):209-15.
30. Wang YM, Li M, Zhao XX, Pan XX, Lu M, Lu J, et al. Effects of continuous care for patients with type 2 diabetes using mobile health application: A randomised controlled trial. International Journal of Health Planning and Management. 2019;34(3):1025-35.
31. Wild SH, Hanley J, Lewis SC, McKnight JA, McCloughan LB, Padfield PL, et al. Supported Telemonitoring and Glycemic Control in People with Type 2 Diabetes: the Telescot Diabetes Pragmatic Multicenter Randomized

Controlled Trial. PLoS medicine. 2016;13(7):e1002098.
32. Yoo HJ, Park MS, Kim TN, Yang SJ, Cho GJ, Hwang TG, et al. A Ubiquitous Chronic Disease Care system using cellular phones and the internet. Diabetic medicine. 2009;26(6):628-35.
33. Zhou PR, Xu LL, Liu XY, Huang JW, Xu WP, Chen WJ. Web-based telemedicine for management of type 2 diabetes through glucose uploads: a randomized controlled trial. International journal of clinical and experimental pathology. 2014;7(12):8848-54.
34. Alghafri TS, Alharthi SM, Al-Farsi Y, Alrawahi AH, Bannerman E, Craigie AM, et al. 'MOVEdiabetes': a cluster randomized controlled trial to increase physical activity in adults with type 2 diabetes in primary health in Oman. BMJ open diabetes research & care. 2018;6(1).
35. Alonso-Domínguez R, Patino-Alonso MC, Sánchez-Aguadero N, García-Ortiz L, Recio-Rodríguez JI, Gómez-Marcos MA. Effect of a multifactorial intervention on the increase in physical activity in subjects with type 2 diabetes mellitus: a randomized clinical trial (EMID Study). European Journal of Cardiovascular Nursing. 2019;18(5):399-409.
36. Asante E, Carter G, McAneney H, Bam V, Sarfo-Kantanka O, Prue G. Nurse-Led Mobile Phone Intervention to Promote Self-Management in Type 2 Diabetes in Ghana: a Randomized Controlled Trial. The science of diabetes self-management and care. 2024:26350106241293113.
37. Bae JH, Park EH, Lee HK, Yoon KH, Won KC, Kim HM, et al. Enhancing Diabetes Care through a Mobile Application: a Randomized Clinical Trial on Integrating Physical and Mental Health among Disadvantaged Individuals. Diabetes & metabolism journal. 2024;48(4):790-801.
38. Boels AM, Vos RC, Dijkhorst-Oei LT, Rutten G. Effectiveness of diabetes self-management education and support via a smartphone application in insulin-treated patients with type 2 diabetes: results of a randomized controlled trial (TRIGGER study). BMJ open diabetes research and care. 2019;7(1).
39. Bonn SE, Hummel M, Peveri G, Eke H, Alexandrou C, Bellocco R, et al. Effectiveness of a Smartphone App to Promote Physical Activity Among Persons With Type 2 Diabetes: Randomized Controlled Trial. Interactive journal of medical research. 2024;13.
40. Callan JA, Sereika SM, Cui R, Tamres LK, Tarneja M, Greene B, et al. Cognitive Behavioral Therapy (CBT) Telehealth Augmented With a CBT Smartphone Application to Address Type 2 Diabetes Self-Management: a Randomized Pilot Trial. The science of diabetes self-management and care. 2022;48(6):492-504.
41. Zamanillo-Campos R, Fiol-deRoque MA, Serrano-Ripoll MJ, Mira-Martinez S, Ricci-Cabello I. Development and evaluation of DiabeText, a personalized mHealth intervention to support medication adherence and lifestyle change behaviour in patients with type 2 diabetes in Spain: a mixed-methods phase II pragmatic randomized controlled clinical trial. International journal of medical informatics. 2023;176:105103.
42. Franc S, Joubert M, Daoudi A, Fagour C, Benhamou PY, Rodier M, et al. Efficacy of two telemonitoring systems to improve glycaemic control during basal insulin initiation in patients with type 2 diabetes: The TeleDiab-2 randomized controlled trial. Diabetes, Obesity and Metabolism. 2019;21(10):2327-32.
43. Han Y, Ye X, Li X, Yang P, Wu Y, Chen L, et al. Comparison of an online versus conventional multidisciplinary collaborative weight loss programme in type 2 diabetes mellitus: a randomized controlled trial. International journal of nursing practice. 2023;29(1):e13126.
44. Heald AH, Roberts S, Albeda Gimeno L, Gilingham E, James M, White A, et al. A Randomised Control Trial to Explore the Impact and Efficacy of the Healum Collaborative Care Planning Software and App on Condition Management in the Type 2 Diabetes Mellitus Population in NHS Primary Care. Diabetes therapy. 2023;14(6):977-88.
45. Hilmarsdottircc E, Sigurdardottirc AK, Arnardottircc RH. A digital lifestyle program to support outpatient

treatment of type 2 diabetes: a randomized controlled trial. Journal of diabetes science and technology. 2020;14(2):A49.

46. Holmen H, Torbjørnsen A, Wahl AK, Jenum AK, Småstuen MC, Årsand E, et al. A mobile health intervention for self-management and lifestyle change for persons with type 2 diabetes, part 2: One-year results from the norwegian randomized controlled trial RENEWING HEALTH. JMIR mHealth and uHealth. 2014;2(4).

47. Huang ZL, Tan E, Lum E, Sloot P, Boehm BO, Car J. A Smartphone App to Improve Medication Adherence in Patients With Type 2 Diabetes in Asia: Feasibility Randomized Controlled Trial. JMIR mHealth and uHealth. 2019;7(9).

48. Iljaž R, Brodnik A, Zrimec T, Cukjati I. E-healthcare for Diabetes Mellitus Type 2 Patients - A Randomised Controlled Trial in Slovenia. Zdravstveno varstvo. 2017;56(3):150-7.

49. Kleinman NJ, Shah A, Shah S, Phatak S, Viswanathan V. Improved Medication Adherence and Frequency of Blood Glucose Self-Testing Using an m-Health Platform Versus Usual Care in a Multisite Randomized Clinical Trial Among People with Type 2 Diabetes in India. Telemedicine journal and e-health. 2017;23(9):733-40.

50. Lee EY, Cha SA, Yun JS, Lim SY, Lee JH, Ahn YB, et al. Efficacy of Personalized Diabetes Self-care Using an Electronic Medical Record-Integrated Mobile App in Patients With Type 2 Diabetes: 6-Month Randomized Controlled Trial. Journal of medical Internet research. 2022;24(7):e37430.

51. Liang G, Jiang H, Huang C, Que X, Tang J, Lu J, et al. Diabetes health management strategy based on internet plus graded diagnosis and treatment strategy. Annals of palliative medicine. 2020;9(6):3915-22.

52. Lu Z, Li Y, He Y, Zhai Y, Wu J, Wang J, et al. Internet-Based Medication Management Services Improve Glycated Hemoglobin Levels in Patients with Type 2 Diabetes. Telemed J E Health. 2021;27(6):686-93.

53. Luo ES, Wan JJ, Su MT, Wang JY, Feng JH, Xie X, et al. Efficacy of a basal insulin dose management smartphone application for controlling fasting blood glucose in patients with type-2 diabetes mellitus: A single-centre, randomised clinical study. Clinical Endocrinology. 2023;99(4):361-9.

54. Di Molfetta S, Laviola L, Natalicchio A, Leonardini A, Cignarelli A, Bonizzoni E, et al. Evaluation of a digital tool supporting therapeutic decision making for the personalized management of patients with type 2 diabetes not treated with insulin: a pilot study. Diabetes research and clinical practice. 2023;203:110836.

55. Poonprapai P, Lerkiatbundit S, Saengcharoen W. Family support-based intervention using a mobile application provided by pharmacists for older adults with diabetes to improve glycaemic control: a randomised controlled trial. International journal of clinical pharmacy. 2022;44(3):680-8.

56. Quinn CC, Shardell MD, Terrin ML, Barr EA, Park D, Shaikh F, et al. Mobile Diabetes Intervention for Glycemic Control in 45- to 64-Year-Old Persons with Type 2 Diabetes. Journal of Applied Gerontology. 2016;35(2):227-43.

57. Riangkam C, Sriyuktasuth A, Pongthavornkamol K, Kusakunniran W, Sriwijitkamol A. Effects of a mobile health diabetes self-management program on HbA1C, self-management and patient satisfaction in adults with uncontrolled type 2 diabetes: a randomized controlled trial. Journal of Health Research. 2022;36(5):878-88.

58. Sachmechi I, Amini M, Salam S, Khan R, Spitznogle A, Belen T. Frequent Monitoring Of Blood Glucose Levels Via A Remote Patient Monitoring System Helps Improve Glycemic Control. Journal of the Endocrine Society. 2023;7:A519-A20.

59. Sokolovska J, Ostrovska K, Pahirko L, Varblane G, Krilatiha K, Cirulnieks A, et al. Impact of interval walking training managed through smart mobile devices on albuminuria and leptin/adiponectin ratio in patients with type 2 diabetes. Physiological Reports. 2020;8(13).

60. Wang J, Cai CY, Padhye N, Orlander P, Zare M. A Behavioral Lifestyle Intervention Enhanced With Multiple-Behavior Self-Monitoring Using Mobile and Connected Tools for Underserved Individuals With Type 2

Diabetes and Comorbid Overweight or Obesity: Pilot Comparative Effectiveness Trial. JMIR mHealth and uHealth. 2018;6(4).
61. Yang Y, Lee EY, Kim HS, Lee SH, Yoon KH, Cho JH. Effect of a Mobile Phone-Based Glucose-Monitoring and Feedback System for Type 2 Diabetes Management in Multiple Primary Care Clinic Settings: cluster Randomized Controlled Trial. JMIR mHealth and uHealth. 2020;8(2):e16266.
62. Yang L, Xu J, Kang C, Bai Q, Wang XY, Du SS, et al. Effects of Mobile Phone Based Telemedicine Management in Patients With Type 2 Diabetes Mellitus: A Randomized Clinical Trial. American Journal of the Medical Sciences. 2022;363(3):224-31.
63. Yin W, Liu Y, Hu H, Sun J, Liu Y, Wang Z. Telemedicine management of type 2 diabetes mellitus in obese and overweight young and middle-aged patients during COVID-19 outbreak: a single-center, prospective, randomized control study. PloS one. 2022;17(9):e0275251.
64. Yu Y, Yan Q, Li H, Li H, Wang L, Wang H, et al. Effects of mobile phone application combined with or without self-monitoring of blood glucose on glycemic control in patients with diabetes: A randomized controlled trial. Journal of diabetes investigation. 2019;10(5):1365-71.
65. Zamanillo-Campos R, Fiol-DeRoque MA, Serrano-Ripoll MJ, Llobera J, Taltavull-Aparicio JM, Leiva A, et al. Impact of an SMS intervention to support type 2 diabetes self-management: diabeText clinical trial. British journal of general practice. 2024.
66. Zhai YK, Yu WJ. A Mobile App for Diabetes Management: Impact on Self-Efficacy Among Patients with Type 2 Diabetes at a Community Hospital. Medical Science Monitor. 2020;26.
67. Zhang PH, Tao XC, Ma YX, Zhang YS, Ma XY, Song HY, et al. Improving the management of type 2 diabetes in China using a multifaceted digital health intervention in primary health care: the SMARTDiabetes cluster randomised controlled trial. Lancet Regional Health-Western Pacific. 2024;49.
68. Avdal EU, Kizilci S, Demirel N. The effects of web-based diabetes education on diabetes care results: A randomized control study. CIN - Computers Informatics Nursing. 2011;29(2):101-6.
69. Spierling Bagsic SR, Padilla Neely OM, Sandoval H, Bastian A, Belasco R, Orendain N, et al. Outcomes of the Dulce Digital-COVID Aware (DD-CA) Discharge Texting Platform for U.S./Mexico-Border Hispanics with Diabetes. Diabetes. 2023;72.
70. Bender MS, Cooper BA, Park LG, Padash S, Arai S. A feasible and efficacious mobile-phone based lifestyle intervention for filipino americans with type 2 diabetes: randomized controlled trial. JMIR diabetes. 2017;2(2):e30.
71. Blioumpa C, Karanasiou E, Antoniou V, Batalik L, Kalatzis K, Lanaras L, et al. Efficacy of supervised home-based, real time, videoconferencing telerehabilitation in patients with type 2 diabetes: a single-blind randomized controlled trial. European journal of physical and rehabilitation medicine. 2023;59(5):628-39.
72. Christensen JR, Laursen DH, Lauridsen JT, Hesseldal L, Jakobsen PR, Nielsen JB, et al. Reversing Type 2 Diabetes in a Primary Care-Anchored eHealth Lifestyle Coaching Programme in Denmark: a Randomised Controlled Trial. Nutrients. 2022;14(16).
73. Connelly J, Kirk A, Masthoff J, MacRury S. A Website to Promote Physical Activity in People With Type 2 Diabetes Living in Remote or Rural Locations: Feasibility Pilot Randomized Controlled Trial. JMIR diabetes. 2017;2(2):e26.
74. Dening J, Mohebbi M, Abbott G, George ES, Ball K, Islam SMS. A web-based low carbohydrate diet intervention significantly improves glycaemic control in adults with type 2 diabetes: results of the T2Diet Study randomised controlled trial. Nutrition & diabetes. 2023;13(1):12.
75. Duruturk N, Özköslü MA. Effect of tele-rehabilitation on glucose control, exercise capacity, physical fitness, muscle strength and psychosocial status in patients with type 2 diabetes: a double blind randomized controlled trial.

Primary care diabetes. 2019;13(6):542-8.
76. Esferjani SV, Naghizadeh E, Albokordi M, Zakerkish M, Araban M. Effectiveness of a mobile-based educational intervention on self-care activities and glycemic control among the elderly with type 2 diabetes in southwest of Iran in 2020. Archives of public health = Archives belges de sante publique. 2022;80(1):201.
77. Gong EY, Baptista S, Russell A, Scuffham P, Riddell M, Speight J, et al. My Diabetes Coach, a Mobile App-Based Interactive Conversational Agent to Support Type 2 Diabetes Self-Management: Randomized Effectiveness-Implementation Trial. Journal of Medical Internet Research. 2020;22(11).
78. Gupta U, Gupta Y, Jose D, Mani K, Jyotsna VP, Sharma G, et al. Effectiveness of a Video-Based Lifestyle Education Program Compared to Usual Care in Improving HbA1c and Other Metabolic Parameters in Individuals with Type 2 Diabetes: an Open-Label Parallel Arm Randomized Control Trial (RCT). Diabetes therapy. 2020;11(3):667-79.
79. Höchsmann C, Müller O, Ambühl M, Klenk C, Königstein K, Infanger D, et al. Novel Smartphone Game Improves Physical Activity Behavior in Type 2 Diabetes. American journal of preventive medicine. 2019;57(1):41-50.
80. Jaipakdee J, Jiamjarasrangsi W, Lohsoonthorn V, Lertmaharit S. Effectiveness of a self-management support program for Thais with type 2 diabetes: evaluation according to the RE-AIM framework. Nursing & health sciences. 2015;17(3):362-9.
81. Welch G, Zagarins SE, Santiago-Kelly P, Rodriguez Z, Bursell SE, Rosal MC, et al. An internet-based diabetes management platform improves team care and outcomes in an urban latino population. Diabetes care. 2015;38(4):561-7.
82. Joshi S, Shamanna P, Dharmalingam M, Vadavi A, Keshavamurthy A, Shah L, et al. Digital Twin-Enabled Personalized Nutrition Improves Metabolic Dysfunction-Associated Fatty Liver Disease in Type 2 Diabetes: results of a 1-Year Randomized Controlled Study. Endocrine practice. 2023;29(12):960-70.
83. Kargarshuroki M, Sadeghian HA, Fatehi F, Martini M, Rahmanian M, Tafti AD. The effect of diabetes training through social networks on metabolic control of individuals with type 2 diabetes; a randomized controlled trial. Journal of preventive medicine and hygiene. 2023;64(4):E499-E506.
84. Kim SH, Kim Y, Choi S, Jeon B. Evaluation of nurse-led social media intervention for diabetes self-management: a mixed-method study. Journal of nursing scholarship : an official publication of Sigma Theta Tau International Honor Society of Nursing. 2022;54(5):569-77.
85. Kim KM, Park KS, Lee HJ, Lee YH, Bae JS, Lee YJ, et al. Efficacy of a New Medical Information system, Ubiquitous Healthcare Service with Voice Inception Technique in Elderly Diabetic Patients. Scientific reports. 2015;5.
86. Kardaş Kin Ö, Türeyen A. The effect of diabetes education based on learning modality in individuals with diabetes incompatible with treatment on compliance and metabolic goals: a randomized controlled trial. Primary care diabetes. 2022;16(1):150-5.
87. Leong CM, Lee TI, Chien YM, Kuo LN, Kuo YF, Chen HY. Social Media-Delivered Patient Education to Enhance Self-management and Attitudes of Patients with Type 2 Diabetes During the COVID-19 Pandemic: randomized Controlled Trial. Journal of medical Internet research. 2022;24(3):e31449.
88. Lorig K, Ritter PL, Laurent DD, Plant K, Green M, Jernigan VBB, et al. Online diabetes self-management program: A randomized study. Diabetes care. 2010;33(6):1275-81.
89. O'Neil PM, Miller-Kovach K, Tuerk PW, Becker LE, Wadden TA, Fujioka K, et al. Randomized controlled trial of a nationally available weight control program tailored for adults with type 2 diabetes. Obesity. 2016;24(11):2269-77.

90. Poppe L, De Bourdeaudhuij I, Verloigne M, Shadid S, Van Cauwenberg J, Compernolle S, et al. Efficacy of a Self-Regulation-Based Electronic and Mobile Health Intervention Targeting an Active Lifestyle in Adults Having Type 2 Diabetes and in Adults Aged 50 Years or Older: two Randomized Controlled Trials. Journal of medical Internet research. 2019;21(8):e13363.
91. Ramadas A, Chan CKY, Oldenburg B, Hussein Z, Quek KF. Randomised-controlled trial of a web-based dietary intervention for patients with type 2 diabetes: changes in health cognitions and glycemic control. BMC public health. 2018;18(1):716.
92. Shah MK, Wyatt LC, Gibbs-Tewary C, Zanowiak JM, Mammen S, Islam N. A Culturally Adapted, Telehealth, Community Health Worker Intervention on Blood Pressure Control among South Asian Immigrants with Type II Diabetes: results from the DREAM Atlanta Intervention. Journal of general internal medicine. 2024;39(4):529-39.
93. Sáenz A, Brito M, Morón I, Torralba A, García-Sanz E, Redondo J. Development and validation of a computer application to aid the physician's decision-making process at the start of and during treatment with insulin in type 2 diabetes: a randomized and controlled trial. Journal of diabetes science and technology. 2012;6(3):581-8.
94. Terkes N, Bektas H, Balci MK. Effect of web-based education intervention on blood glucose control, self-care and quality of life in patients with type 2 diabetes: a single-blinded randomized controlled trial. International journal of nursing practice. 2024;30(6):e13298.
95. Terkes N, Aksu NT, Yamac SU. The effect of an online-supervised exercise program in older people with diabetes on fasting blood sugar, psychological resilience and quality of life: a double blind randomised controlled trial. International journal of older people nursing. 2023;18(5):e12564.
96. Warren R, Carlisle K, Mihala G, Scuffham PA. Effects of telemonitoring on glycaemic control and healthcare costs in type 2 diabetes: a randomised controlled trial. Journal of telemedicine and telecare. 2018;24(9):586-95.
97. Ye HJ, Lin L, Zhong DM, Chen P, He XQ, Luo ZR, et al. The impact of telehealth education on self-management in patients with coexisting type 2 diabetes mellitus and hypertension: a 26-week randomized controlled trial. Journal of endocrinological investigation. 2024;47(9):2361-9.
98. Asante E, Bam V, Diji AKA, Lomotey AY, Owusu Boateng A, Sarfo-Kantanka O, et al. Pilot Mobile Phone Intervention in Promoting Type 2 Diabetes Management in an Urban Area in Ghana: A Randomized Controlled Trial. The Diabetes educator. 2020;46(5):455-64.
99. Benson GA, Sidebottom A, Hayes J, Miedema MD, Boucher J, Vacquier M, et al. Impact of ENHANCED (diEtitiaNs Helping pAtieNts CarE for Diabetes) Telemedicine Randomized Controlled Trial on Diabetes Optimal Care Outcomes in Patients with Type 2 Diabetes. Journal of the Academy of Nutrition and Dietetics. 2019;119(4):585-98.
100. Farmer A, Bobrow K, Leon N, Williams N, Phiri E, Namadingo H, et al. Digital messaging to support control for type 2 diabetes (StAR2D): a multicentre randomised controlled trial. Bmc Public Health. 2021;21(1).
101. Döbler A, Herbeck Belnap B, Pollmann H, Farin E, Raspe H, Mittag O. Telephone-delivered lifestyle support with action planning and motivational interviewing techniques to improve rehabilitation outcomes. Rehabilitation psychology. 2018;63(2):170-81.
102. ElizabethG. Eakin. Living Well With Diabetes: 24-Month Outcomes From a Randomized Trial ofTelephoneDelivered Weight Loss and Physical Activity Intervention to Improve Glycemic Control. Diabetes care. 2014:2177–85.
103. Fortmann AL, Gallo LC, Garcia MI, Taleb M, Euyoque JA, Clark T, et al. Dulce digital: An mHealth SMS based intervention improves glycemic control in hispanics with type 2 diabetes. Diabetes care. 2017;40(10):1349-55.
104. Graziano JA, Gross CR. A randomized controlled trial of an automated telephone intervention to improve

glycemic control in type 2 diabetes. ANS Advances in nursing science. 2009;32(3):E42-57.
105. Haghighinejad H, Liaghat L, Malekpour F, Jafari P, Taghipour K, Rezaie M, et al. Comparing the effects of SMS-based education with group-based education and control group on diabetes management: a randomized educational program. BMC primary care. 2022;23(1):209.
106. Hoda F, Arshad M, Khan MA, Kohli S, Kareem S, Akhtar M, et al. Impact of a mHealth Intervention in Type 2 Diabetes Mellitus Patients: a Randomized Clinical Trial. SN comprehensive clinical medicine. 2023;5(1).
107. Kasar KS, Asiret GD, Yilmaz CK, Canlar S. The effect of model-based telephone counseling on HbA1c and self-management for individuals with type 2 diabetes: A randomized controlled trial. Primary care diabetes. 2022;16(1):41-8.
108. Kim K, Yun JS, Lee J, Yang Y, Lee M, Ahn YB, et al. Effectiveness of a Social Networking Site Based Automatic Mobile Message Providing System on Glycemic Control in Patients with Type 2 Diabetes Mellitus. Endocrinology and metabolism (Seoul, Korea). 2024;39(2):344-52.
109. Lauffenburger J, Ghazinouri R, Jan S, Makanji S, Ferro C, Lewey J, et al. Effect of a novel pharmacist-delivered behavioral intervention for patients with poorly-controlled diabetes: The enhancing outcomes through goal assessment and generating engagement in diabetes mellitus pragmatic, database-randomized controlled trial. Pharmacoepidemiology and Drug Safety. 2019;28:223.
110. Liu XY, Wang XH, Xie MX, Cao LL. Application of the integrated data platform combined with dietary management for adults with diabetes: A prospective randomized controlled trial. Journal of diabetes investigation. 2024;15(11):1548-55.
111. Middleton T, Constantino M, McGill M, D'Souza M, Twigg SM, Wu T, et al. An Enhanced SMS Text Message-Based Support and Reminder Program for Young Adults With Type 2 Diabetes (TEXT2U): randomized Controlled Trial. Journal of medical Internet research. 2021;23(10):e27263.
112. Mons U, Raum E, Krämer HU, Rüter G, Rothenbacher D, Rosemann T, et al. Effectiveness of a supportive telephone counseling intervention in type 2 diabetes patients: randomized controlled study. PloS one. 2013;8(10):e77954.
113. Moreira AM, Rados DV, de Farias CB, Coelli S, de Almeida Faller L, dos Santos LF, et al. Effects of nurse tele support via telephone calls on transition between specialized and primary care in type 2 diabetes mellitus patients: a CONSORT-compliant randomized clinical trial. Endocrine. 2024.
114. Peimani M, Rambod C, Omidvar M, Larijani B, Ghodssi-Ghassemabadi R, Tootee A, et al. Effectiveness of short message service-based intervention (SMS) on self-care in type 2 diabetes: a feasibility study. Primary care diabetes. 2016;10(4):251-8.
115. Ramirez M, Wu S. Phone Messaging to Prompt Physical Activity and Social Support Among Low-Income Latino Patients With Type 2 Diabetes: a Randomized Pilot Study. JMIR diabetes. 2017;2(1):e8.
116. Sarayani A, Mashayekhi M, Nosrati M, Jahangard-Rafsanjani Z, Javadi M, Saadat N, et al. Efficacy of a telephone-based intervention among patients with type-2 diabetes; a randomized controlled trial in pharmacy practice. International journal of clinical pharmacy. 2018;40(2):345-53.
117. Shahid M, Mahar SA, Shaikh S, Shaikh ZUD. Mobile phone intervention to improve diabetes care in rural areas of Pakistan: A randomized controlled trial. Journal of the College of Physicians and Surgeons Pakistan. 2015;25(3):166-71.
118. Hérica Cristina Alves de Vasconcelos JCGLN. Telecoaching programme for type 2 diabetes control: a randomised clinical trial. Diabetes. 2015;64:A188.
